# Structure-Aware Calibration Refinement for Monocular Roadside Cameras Using a Horizontal Ground-Coordinate Objective and Road-Patch Phase Correlation

**DOI:** 10.3390/s26196227

**Published:** 2026-09-30

**Authors:** Ikhyeon Jo, Gooman Park

**Affiliations:** 1 Department of Smart ICT Convergence Engineering, Seoul National University of Science and Technology, 232 Gongneung-ro, Nowon-gu, Seoul 01811, Republic of Korea; ihjo@signtelecom.com; 2 Signtelecom Co., Ltd., Seoul 07299, Republic of Korea

**Keywords:** monocular roadside camera, camera-to-ground mapping, horizontal ground-coordinate objective, ground-plane homography, phase correlation, road-surface patch, time-varying view correction

## Abstract

**Highlights:**

**What are the main findings?**

- Across 14 cameras, the ground-coordinate objective reduced in-sample all-point MHPE by 24.4% at the same control points used to fit the mapping. The held-out overall LOO MHPE decreased from 0.398 to 0.348 m, with improvement in seven out of 14 cameras.
- On the original 40 frames, Phase–Affine had a lower observed MHPE under the present annotations (0.392 versus 0.462 m for native-resolution patch DISK + LightGlue–Affine) and a lower measured mean core runtime (9.858 versus 106.094 ms).

**What are the implications of the main findings?**

- The horizontal-coordinate objective improves the fit to the complete surveyed control-point set; complementary held-out evaluation identifies the extent and geometry-dependence of predictive improvement.
- Road-patch phase correlation provides a low-cost correction option under the evaluated protocol and hardware. The small observed accuracy difference requires cautious interpretation because annotation repeatability was not formally measured.

**Abstract:**

Monocular roadside cameras require accurate image-to-ground mapping and correction of view changes caused by mounting-structure deformation. We combine a horizontal ground-coordinate calibration objective with road-patch phase correlation and affine correction on selected approximately planar road sections with an approximately constant grade. The initial calibration uses 190 reviewed image–global navigation satellite system (GNSS) correspondences from 14 equally weighted cameras at seven sites. With all available control points used for fitting, the camera-balanced mean horizontal position error (MHPE) at those points decreases from 0.259 to 0.196 m (24.4%), and the all-point root mean square error (RMSE) decreases for every camera. This is in-sample fitting performance at the same control points used to estimate the mapping, not independent predictive accuracy. Complementary landmark-level leave-one-out (LOO) evaluation measures prediction at withheld points: the interior LOO decreases from 0.331 to 0.318 m and the exterior LOO from 0.445 to 0.384 m. Overall LOO decreases from 0.398 to 0.348 m, with improvement in seven out of 14 cameras, indicating geometry-dependent predictive benefit. On the original 40 correction frames, Phase–Affine has a lower observed MHPE under the present annotations: 0.392 m, compared with 0.462 m for native-resolution patch DISK + LightGlue with direct robust affine fitting; both yield 40/40 finite evaluations. Their measured mean core times are 9.858 and 106.094 ms, respectively. A 92-frame hourly experiment examines six approved patch centers, five sizes, normalized cross-correlation (NCC) thresholds, patch subsets and transformation models, retaining large finite errors and separately reporting missing transforms. Full-image learned features are more accurate than unfiltered 150 px Phase–Affine on these hourly sequences. The results demonstrate improved in-sample calibration fit and a lower measured core runtime for phase-based correction in the evaluated patch comparison. The small observed MHPE difference is interpreted cautiously because single-annotator repeatability was not formally measured. Held-out and sensitivity analyses delimit these benefits rather than establish uniform superiority across cameras, patch configurations or operating conditions.

## 1. Introduction

As automated and cooperative-driving technologies continue to expand, infrastructure-based cooperative perception has become increasingly important for compensating for the limited sensing range and occlusion of onboard sensors by collecting and sharing surrounding traffic information [1]. At intersections and on urban roads in particular, infrastructure sensors can observe traffic participants in vehicle blind spots and at long distances, thereby extending the perception range of individual vehicles. Among the available modalities, roadside cameras can continuously observe broad road areas from elevated positions and wide fields of view, while providing rich visual information at a comparatively low deployment cost; they have therefore become a major infrastructure sensing modality [2,3]. Camera-based infrastructure perception has already expanded from object detection to localization, tracking, and multi-camera fusion, demonstrating the feasibility of continuous operation in real-world environments [3]. As these applications expand, accurate camera calibration for linking image detections to physical road coordinates will become increasingly important.

The accuracy of this image-to-ground mapping can degrade at two distinct stages. The first is the initial calibration, which establishes the absolute image-to-ground relationship from a reference image. The second is the viewpoint change that occurs after installation when temperature variations, loads, wind, or small external forces deform the supporting structure. Even when an accurate mapping is obtained in the reference image, a small subsequent change in camera orientation causes the original homography [4] to no longer correspond precisely to the current image. In distant image regions with strong perspective effects, a displacement of only a few pixels can translate into several meters of ground-position error.

Initial calibration has commonly been performed by estimating a homography or extrinsic parameters from lanes, vanishing points, surveyed landmarks, or GNSS correspondences [5,6,7,8]. The pixel reprojection error commonly minimized in this process is suitable for quantifying image-domain alignment; however, under perspective projection, the same pixel error corresponds to different ground distances depending on its image location. Moreover, even when GNSS ground points are measured precisely, manual selection of their corresponding image locations can retain pixel-level uncertainty because of line thickness, blur, compression, and operator clicking judgment; this study therefore models image-point selection uncertainty using 1–2 px perturbations. The coordinate system in which the residual is defined is not merely a numerical optimization choice; it determines whether the objective is aligned with the error relevant to the deployed service.

Feature-based registration methods such as SIFT, ORB, DISK, and LightGlue can be used to correct viewpoint changes after installation [9,10,11,12]. For roadside cameras, prior studies have corrected homography changes caused by thermal deformation and vibration of the supporting structure through lane-marking redetection, high-definition-map targets, or learned feature matching between reference and current images [13,14,15]. However, full-image correspondences may arise from trees, signs, and buildings at depths different from the road plane, as well as from dynamic objects such as vehicles and pedestrians. Consequently, the dominant transform selected by a robust estimator may appear consistent over the entire image but differ from the correction transform required for the road plane. Learned features and matchers are robust to illumination changes but incur substantial computational cost and may introduce unnecessary flexibility for a fixed camera undergoing only small viewpoint changes. Phase correlation can estimate translation within a restricted patch at low cost [16,17], but it must be combined with stable road-plane structures and an appropriate transformation model.

This study presents a horizontal-coordinate calibration objective and lightweight road-patch Phase–Affine correction for roadside image-to-ground mapping. The initial-calibration comparison treats 14 cameras at seven sites and their 190 reviewed correspondences as one evaluation set. All-point evaluation assesses the fitted calibration when the complete available control-point set is used, while complementary interior and exterior LOO assess prediction within and beyond the remaining point support. A matched 1–2 px perturbation study examines sensitivity on the same 14 cameras. The correction study evaluates accuracy and computation against feature-based methods under the original 40-frame protocol, including direct-match and native-resolution learned-feature baselines. A 92-frame hourly experiment with revised visible-landmark annotations examines patch size, number, layout, selection and model choice. Failure-inclusive summaries preserve adverse outcomes and define the conditions of the observed advantages. All positional errors use horizontal east–north coordinates rather than full 3D distance.

## 2. Related Work

### 2.1. Traffic-Scene Camera Calibration

Because installing large checkerboards in roadside scenes is difficult, roadside cameras have been calibrated using in-scene cues such as lanes, road boundaries, vanishing points, vehicle trajectories, and GNSS measurements. Masoud and Papanikolopoulos recovered intrinsic and extrinsic parameters from geometric elements in traffic scenes, including lanes, pavement markings, and vertical structures [5]. You and Zheng proposed a roadside-camera calibration method that uses vanishing points along lane and vertical directions and combines repeated observations through least-squares estimation [6]. These studies establish road geometry as a useful prior, but they primarily address absolute calibration at the time of installation.

More recently, image-to-ground mappings have also been estimated directly from GNSS-tagged vehicle trajectories or surveyed landmarks. D’Amicantonio et al. represented the topological structure of an intersection and synthetic-view images as a graph and automated homography estimation through learning [7]. Ojala et al. estimated a direct image-to-ground homography from GNSS–image correspondences and used metric ground-plane distances for RANSAC inlier classification and localization evaluation [8]. Because these distances are expressed in meters on the road plane, they quantify the physical localization error presented to downstream road-traffic applications rather than only image-domain alignment. Thus, direct image-to-ground mapping and metric ground-plane evaluation have been addressed in prior work. The focus of this study is instead to compare pixel reprojection and horizontal ground-coordinate objectives directly using identical image–GNSS correspondences and to quantify their effects on final horizontal-position error through all-point fitting assessment, complementary landmark-level leave-one-out evaluation and 1–2 px correspondence-perturbation experiments.

### 2.2. Camera-Mapping Correction Under Structural Deformation and Feature Matching

When deformation of a supporting structure changes the camera viewpoint, a two-dimensional correction transform can be estimated from correspondences between the reference and current images. SIFT provides local gradient descriptors that are robust to scale and rotation changes [9], whereas ORB reduces computation by combining FAST with rotation-aware BRIEF [10]. More recent learned features such as DISK [11] and attention-based matchers such as LightGlue [12] provide strong correspondence performance under substantial illumination and appearance changes. Nevertheless, accurate full-image correspondences do not necessarily yield the correct road-plane correction transform. Structures at different depths, non-rigid vegetation changes, vehicle occlusion, and visual overlays may cause a robust estimator to select a dominant transform that differs from the road-plane transform.

The viewpoint of an installed roadside camera may change because of thermal deformation of its supporting structure, foundation changes, and vibration. During the construction of a large-scale traffic-tracking dataset, Gloudemans et al. showed that the so-called sunflower effect caused by thermal pole deformation induces ground-homography errors; they updated the homography through SIFT–FLANN-based coarse alignment and lane-marking edge redetection [13]. Strand et al. combined correspondence targets derived from a high-definition map with image stabilization to develop a self-correcting calibration architecture for both rapid vibration and slow orientation changes [14]. Fleck et al. combined DISK–LightGlue matching, manual masking of tree crowns, detector-based filtering of moving objects, and homography estimation, and evaluated adaptive calibration using relative world-plane projection MHPE [15]. Together, these works establish time-dependent roadside-camera correction and road-plane-relevant correspondence selection as existing research problems, while highlighting the relevance of metric ground-plane evaluation at both the initial-mapping and adaptive-correction stages [8,15]. Without relying on high-definition-map redetection or global learned features, the present study focuses on a lightweight configuration that combines whole-patch displacement measurements from a small, predefined set of stable road-surface structures as equally weighted patch-center correspondences in a low-complexity affine model. In particular, Fleck et al. evaluate adaptive correction relative to ground positions obtained by projecting reference-image landmarks through an existing calibration [15]. That relative drift metric differs from our held-out GNSS-coordinate error, so their centimeter-scale adaptive result is not numerically comparable with our absolute horizontal landmark errors. Their four-day sequence also covers conditions beyond our per-camera daily sequences. Strand et al. address an HD-map-supported calibration and stabilization architecture, including fast vibration and slow drift [14]; the present hourly experiment does not evaluate transient response or continuous online tracking. We do not claim the first use of metric ground errors, reference-image correction or road-relevant feature selection. Our comparison concerns the calibration loss and the trade-off between sparse-feature and whole-patch measurements.

### 2.3. Phase Correlation and Road-Plane Patches

Phase correlation is a frequency-domain registration method that identifies the correlation peak corresponding to translation by inverse-transforming the normalized cross-power spectrum of two images [16]. It reduces sensitivity to changes in mean brightness and can be implemented efficiently using the fast Fourier transform (FFT), making it suitable for estimating small image displacements. Toikka et al. compensated for translation, rotation, and scale changes in roadside-camera images using frequency-domain registration over the full image and transferred a road-segmentation label from one frame to images captured at other times [17]. Applying phase correlation to roadside-camera viewpoint changes is therefore not itself the novelty of this study. Rather than registering the full image with a single transform, we measure local translation independently within each road-plane patch defined in the reference image, convert each measurement into one equally weighted patch-center correspondence, and estimate a low-complexity affine current-to-reference correction. We further evaluate correction performance using GNSS-linked horizontal ground-coordinate error rather than label overlap and compare ORB, SIFT, DISK + MNN, and DISK + LightGlue under the same patch support and the same affine model.

## 3. Materials and Methods

### 3.1. Image-to-Ground Mapping and Coordinate Transformation

Let the homogeneous pixel coordinate in the initially calibrated reference image *I*_0_ be *p*_0_ = [*u*, *v*, 1]^T^, and let the homogeneous ground coordinate on the road plane be *x* = [*X*, *Y*, 1]^T^. The image-to-ground homography *H*_0_^W^ of the reference image satisfies

*x* ∼ *H*_0_*^W^ p*_0_ (1)

Define *C_t_* as the correction transform from the current image *I_t_* at time *t* to the reference image. A point *p_t_* in the current image is transformed into the reference-image coordinate system as

*p*_0_ ∼ *C_t_ p*_t_ (2)

The camera-to-ground mapping at time *t* is therefore

*H_t_^W^* = *H*_0_*^W^ C_t_*,  *x* ∼ *H_t_^W^ p_t_* (3)

The matrix-product order follows from defining *C_t_* in the current-to-reference direction. In the implementation, the outputs of all baselines and the proposed method are expressed in this same current-to-reference direction.

### 3.2. Pixel-Reprojection and Horizontal Ground-Coordinate Objectives

For the horizontal ground-coordinate objective, GNSS measurements are converted to a local ENU frame and represented by horizontal coordinates *q_i_* = [*E_i_*,*N_i_*,1]^T^. The study is restricted to points on road sections selected as approximately planar with an approximately constant grade. A planar road section need not be horizontal: its horizontal east–north coordinates provide a planar parameterization, so a homography can be fitted directly from image–horizontal-coordinate correspondences without individual point heights. Optimization residuals are computed in the east–north plane; height is not included in the objective. This does not assume that the actual elevations of all road points are zero. The metric is horizontal position error, not distance along the road surface. Image coordinates are represented as *p_i_* = [*u_i_*, *v_i_*, 1]^T^, and π(·) denotes homogeneous-coordinate normalization.

The 2D image-reprojection objective estimates a ground-to-image homography, *G*:

*J*_2D_(*G*) = *Σ_i_* ‖*p_i_* − *π*(*Gq_i_*)‖_2_^2^

Ground coordinates are then predicted using *G*^−1^. By contrast, the horizontal ground-coordinate objective directly estimates an image-to-ground homography, *H*:

*J*_ground_(*H*) = *Σ_i_* ‖*q_i_* − *π*(*Hp_i_*)‖_2_^2^

Both formulations use the same correspondences and the same eight-degree-of-freedom homography; they differ only in the coordinate system of the optimization residual. The former minimizes squared pixel error, whereas the latter minimizes squared metric error. Accordingly, *J*_ground_ is directly aligned with the horizontal-position error relevant to cooperative-driving infrastructure.

### 3.3. Structure-Aware Road Patches

Correction patches are defined as rectangular regions on the road plane in the reference image. Lane boundaries, directional arrows, diamond markings, stop lines, and persistent pavement texture are prioritized, whereas trees, signs, traffic lights, lamp posts, vertical curb faces, and visual-overlay regions are excluded. Each patch is fixed once in the reference image, and the same coordinate region is used in every current image. In the original 40-frame comparison, four road patches are used for each camera, with their centers distributed across image depth and lateral position so that they are not concentrated on one side of the image or along a single line. The separate sensitivity extension in Section 3.14 uses six newly approved centers per camera and does not replace the original patch configuration.

The purpose of this structural constraint is not to increase the number of correspondences but to restrict measurement support to locations relevant to ground mapping. Displacement is computed independently in each patch; the fixed and displaced patch centers are then paired as correspondences, and an image-level affine correction is estimated from all spatially distributed correspondences.

### 3.4. Patch-Wise Displacement Estimation by Phase Correlation

Let *f*_0,*k*_ and *f_t_*_,*k*_ denote the reference and current grayscale patches, respectively, for patch *k* of a camera. After subtracting their means and applying a two-dimensional Hann window, define *F*_0,*k*_ = ℱ{*f*_0,*k*_} and *F_t_*_,*k*_ = ℱ{*f_t_*_,*k*_}. The normalized cross-power spectrum, where * denotes complex conjugation, is

*R_k_*(*ω*) = *F_t,k_*(*ω*)*F*_0,k_^*^(*ω*)/|*F_t_*_,*k*_(*ω*)*F*_0,k_^*^(*ω*)| (4)

The maximum peak of its inverse Fourier transform represents the location to which the reference pattern has moved in the current patch:

*r_k_* = *ℱ*^−1^{*R_k_*},   *Δ_k_* = (*dx_k_*, *dy_k_*) = *arg max*
*r_k_* (5)

Parabolic interpolation over three samples around the integer peak is applied separately along the *x* and y directions to obtain subpixel offsets. Because of the cyclic property of the FFT, a peak beyond half the patch width or height is converted to a negative displacement. The returned *Δ_k_* follows the current-minus-reference convention; therefore, with reference patch center *c*_0,*k*_, the current center is *c_t_*_,*k*_ = *c*_0,*k*_ + *Δ_k_*.

### 3.5. Affine Correction from Patch Displacements

In the original 40-frame comparison, the defined patch-center correspondences are used without a separate patch-quality threshold or update-rejection rule. Section 3.14 separately investigates NCC-based patch selection; its results are not pooled with the original comparison.

Given the set *K_t_* of patch-center correspondences computed from the road patches, the reference-to-current affine transform *A_t_* is estimated by solving

A_t_^*^ = arg min *_A_ Σ_k_*∈*K_t_* ‖*c_t_*_,*k*_ − *π*(*Ac*_0,*k*_)‖_2_^2^ (6)

*At* least three non-collinear patches are required for affine fitting. The final correction is defined as *C_t_* = *A_t_*^−1^, maintaining the current-to-reference direction used by all methods. The corrected camera-to-ground mapping is therefore *H_t_*^W^ = *H*_0_^W^C_t_. Compared with a homography, the lower-complexity six-parameter 2D affine model permits small translations, rotations, scale changes, and shear caused by slight deformation of the supporting structure, while limiting amplification of patch noise through projective terms.

### 3.6. Initial-Calibration Data and Coordinate System

**Figure 1:**
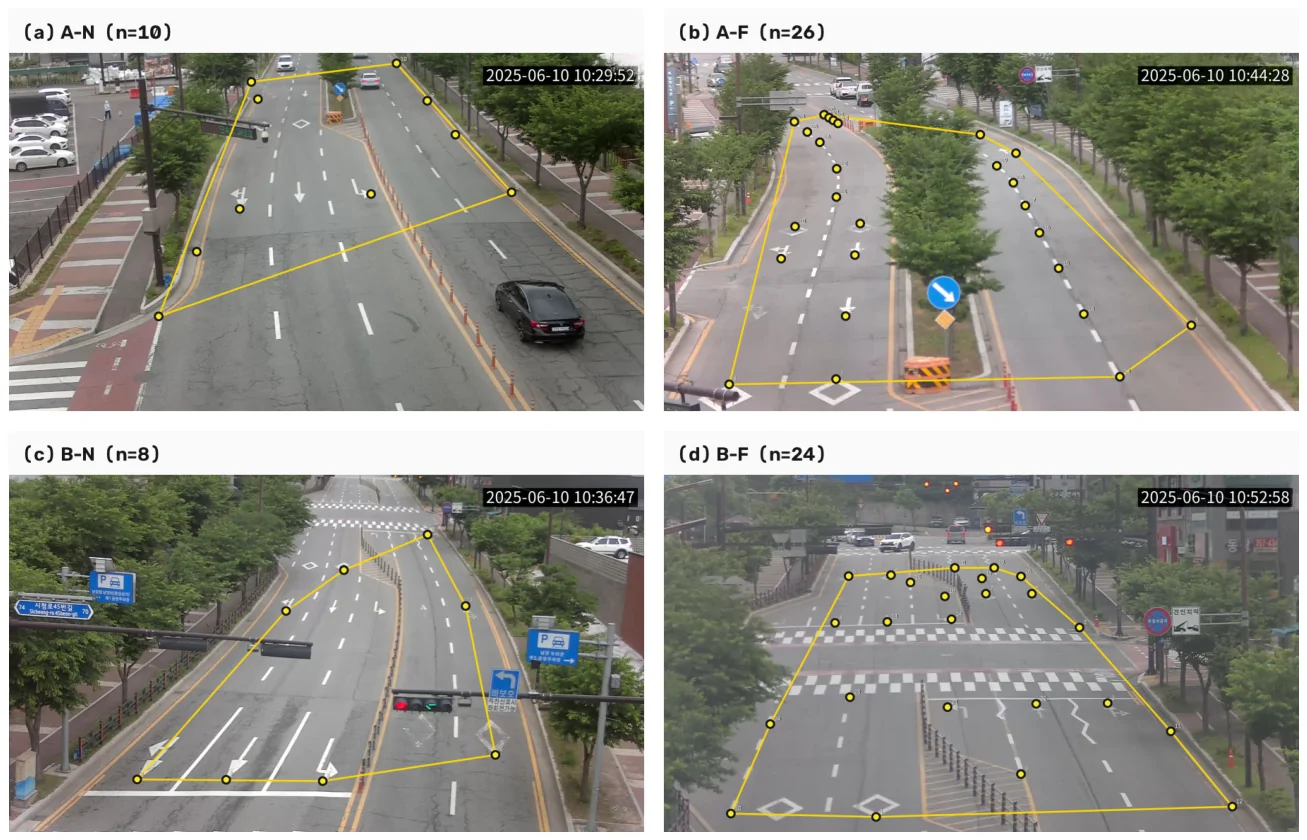
Reference-image calibration-point layouts for the four cameras also used in the temporal correction experiments: (**a**) A-N, 10 points; (**b**) A-F, 26 points; (**c**) B-N, 8 points; and (**d**) B-F, 24 points. Yellow dots are GNSS correspondences and yellow outlines are image-coordinate convex hulls. The complete 14-camera static results appear in Table 1.

The initial-calibration dataset contains 190 reviewed image–GNSS correspondences from 14 cameras at seven installation sites. Paired near- and far-range views are identified as A-N/A-F, B-N/B-F, and 22-N/22-F through 26-N/26-F. Each pair shares an installation site and differs in magnification; N and F identify the supplied nominal near- and far-range groups rather than measured point distances. All 14 cameras have equal status in the analysis, with no separate original-versus-additional cohort comparison. Point counts range from 8 to 26 and are reported in Table 1. WGS84 coordinates are converted to local ENU coordinates, and only east–north coordinates enter the objectives and evaluation. Figure 1 illustrates the four cameras for which temporal correction data are also available; the static evaluation includes all 14 cameras.

Ground-reference coordinates were collected with the sensor-integrated GNSS terminal listed in [18], using the LG U+ RTK correction service (LG Uplus Corp., Seoul, Republic of Korea). The operator checked normal terminal operation and correction-signal reception and recorded coordinates after confirming an RTK Fixed solution. The product information identifies a u-blox F9(P/R)-family engine (u-blox AG, Thalwil, Switzerland) and an external multiband antenna supporting L1/L2/E5b/B2I, and it specifies a nominal stationary-point RTK positioning precision of 1 cm + 1 ppm under open-sky conditions [18]. This is a published equipment specification, not an independently measured uncertainty for the surveyed points in this study. Surveyed physical corners or road-marking boundaries were associated with identifiable image landmarks, and the same landmark identities were retained across frames. RTK Fixed status does not remove antenna-placement or image-annotation uncertainty; the 1–2 px experiment assesses sensitivity to image-point selection, not GNSS uncertainty.

### 3.7. Objective-Function Comparison and Evaluation

Three complementary evaluations use the same reviewed correspondences and eight-degree-of-freedom homography for both objectives. All-point evaluation first assesses the fitted calibration using the complete available control-point set: all n correspondences estimate the mapping, and the same n points evaluate it. This directly measures how accurately the fitted mapping reproduces its surveyed control points, rather than independent predictive accuracy. Interior and exterior LOO then provide complementary held-out evaluation of interpolation and extrapolation. In landmark-level LOO, point i is withheld before fitting to the other n − 1 points. A fold is interior LOO if the withheld image point lies inside or on the convex hull of the remaining image points, and exterior LOO otherwise. The classification is geometric, uses image coordinates rather than observed errors, and is identical for both objectives. It distinguishes interpolation from extrapolation relative to the remaining support, not points inside versus outside the image. The pixel objective fits a ground-to-image homography G and evaluates G^−1^; the ground objective directly fits an image-to-ground homography H. Horizontal error is the Euclidean distance between the predicted and GNSS east–north coordinates. MHPE first averages point errors within each camera and evaluation category, then weights all 14 camera means equally. The 97 interior and 93 exterior folds together exhaust all 190 LOO folds; every camera contributes to both categories. Overall LOO combines all folds within each camera before the camera average and is not the unweighted mean of the two category summaries.

### 3.8. Sensitivity Experiment with Perturbed Image Correspondences

Even when ground coordinates are measured using high-precision GNSS, the corresponding image location must be selected by an operator observing a lane, pavement marking, or structural boundary. Line thickness, perspective shrinkage, blur, compression, zoom level, and clicking judgment can introduce pixel-level differences in the selected image location. In informal annotation work, the same annotator reported that differences observed when repeatedly marking the same image were within approximately 2 px. This experience provides a practical motivation for examining perturbations on the order of 1–2 px; it is not a formal estimate of annotation accuracy or an empirically measured error distribution. We therefore use random 1–2 px perturbations as a sensitivity test at this scale. The uniform displacement magnitudes and random directions specified below are simulation assumptions, not a fitted model of operator error.

For each camera and repetition, an independent direction uniformly distributed over [0, 2π) and a magnitude uniformly distributed over [1,2] px are sampled for every image point. This perturbs all training points simultaneously, rather than moving one training point at a time. In each LOO fold, the held-out point is excluded from fitting and evaluated at its original reviewed image location; the remaining points use their sampled perturbations. The same perturbation realization is shared by both objectives and reused across folds within the repetition. Ground coordinates remain fixed. After fixing the random-number generator seed at 20,260,817, we generated 1000 distinct random correspondence perturbations of 1–2 px and performed repeated evaluations on all 14 cameras. Camera-specific deterministic random streams use the source camera identifiers. Every repetition computes camera-wise LOO MHPE and the equally weighted 14-camera mean.

### 3.9. Time-Distributed Images for Mounting-Structure-Induced View Correction

The mounting-structure-induced view correction experiment used 10 images per camera, for a total of 40 images, selected from images captured at approximately one-hour intervals. To avoid concentration within a particular period of the day, each camera contributed two late-night, two dawn, two morning, two midday, and two afternoon images. Final current-image evaluation coordinates were reviewed and corrected by one annotator using a consistent correspondence criterion. The surveyed landmarks were associated with recognizable physical corners or marking boundaries, and the same landmark identities were maintained across frames. Human review does not imply error-free image coordinates; the informal repeat-placement observation and synthetic uncertainty experiment are described separately in Section 3.8.

The four patches defined in each reference image were applied at the same rectangular coordinates in all current images. Frame-wise evaluation points were the current-image coordinates corresponding to the original calibration landmarks, and the original GNSS ground coordinates remained the reference values. Thus, the correction transform estimated by every method was applied to identical evaluation points and identical ground reference coordinates, and horizontal ground-position errors were compared.

### 3.10. Compared Methods and Implementation Settings

The comparison includes full-image feature–homography pipelines, patch-restricted feature–homography pipelines, and an equal-weight patch-center affine protocol. The last protocol gives every patch one representative displacement, intentionally preventing texture-rich patches from dominating. It evaluates that representation rather than an unrestricted best implementation of each matcher. Section 3.17 therefore adds affine fits directly from individual matches and independently extracted native-resolution DISK patches. Feature–patch fusion is not included. ORB and SIFT use descriptor-ratio matching; DISK + MNN uses mutual cosine-similarity matches and DISK + LightGlue uses the official pretrained DISK matcher.

Runtime was measured separately using the same 40-frame manifest as the accuracy experiment and a fixed-reference operating condition. Each camera’s reference-image representation was computed once and excluded as an offline cost. Core time was measured from the decoded current image and included preprocessing, CPU-to-GPU transfer, feature extraction or FFT computation, matching, reduction to a representative patch displacement, and affine fitting. After five warm-up runs for each method and camera, each frame was repeated 10 times and the median was recorded; GPU stages were synchronized. Because the implementations use a mixture of CPU and GPU execution, runtime is interpreted as computational cost under the experimental environment equipped with an NVIDIA RTX 3090.

The original fixed-reference runtime experiments were executed with Python 3.12.13, OpenCV 5.0.0, NumPy 2.5.2, PyTorch 2.12.1+cu132, and CUDA 13.2 on an NVIDIA RTX 3090. The archived environments for the strengthened-baseline, direct-fit runtime, and hourly extensions record Python 3.12.14 with the same listed library and CUDA versions. The official LightGlue implementation was fixed at commit eb42fee2d71449efb0aa5c10549752b5d75384d8; run-specific environment records are provided in Supplementary Materials S1. DISK extraction used at most 4096 keypoints.

### 3.11. Full-Image and Patch-Scope Comparison for Feature Methods

To compare full-image feature matching with matching restricted to road patches, both conditions were implemented for ORB, SIFT, DISK + MNN, and DISK + LightGlue. In the patch condition for ORB and SIFT, features were detected and described only within road patches in the reference image. In the current image, each region was expanded by 40 px to accommodate the expected displacement, and the same procedure was applied. Both conditions used identical matching settings and the USAC_MAGSAC homography estimator.

In the original DISK patch configuration, features are extracted once from the full image and subset by patch membership. This avoids separate crop calls but can allocate few keypoints to a small road region, especially after global resizing. It is an implementation choice rather than a demonstrated universal efficiency advantage. Section 3.17 separately evaluates native-resolution independent crop extraction and benchmarks the resulting stronger baselines. Neither configuration fuses a global correction with patch correction.

**Figure 2:**
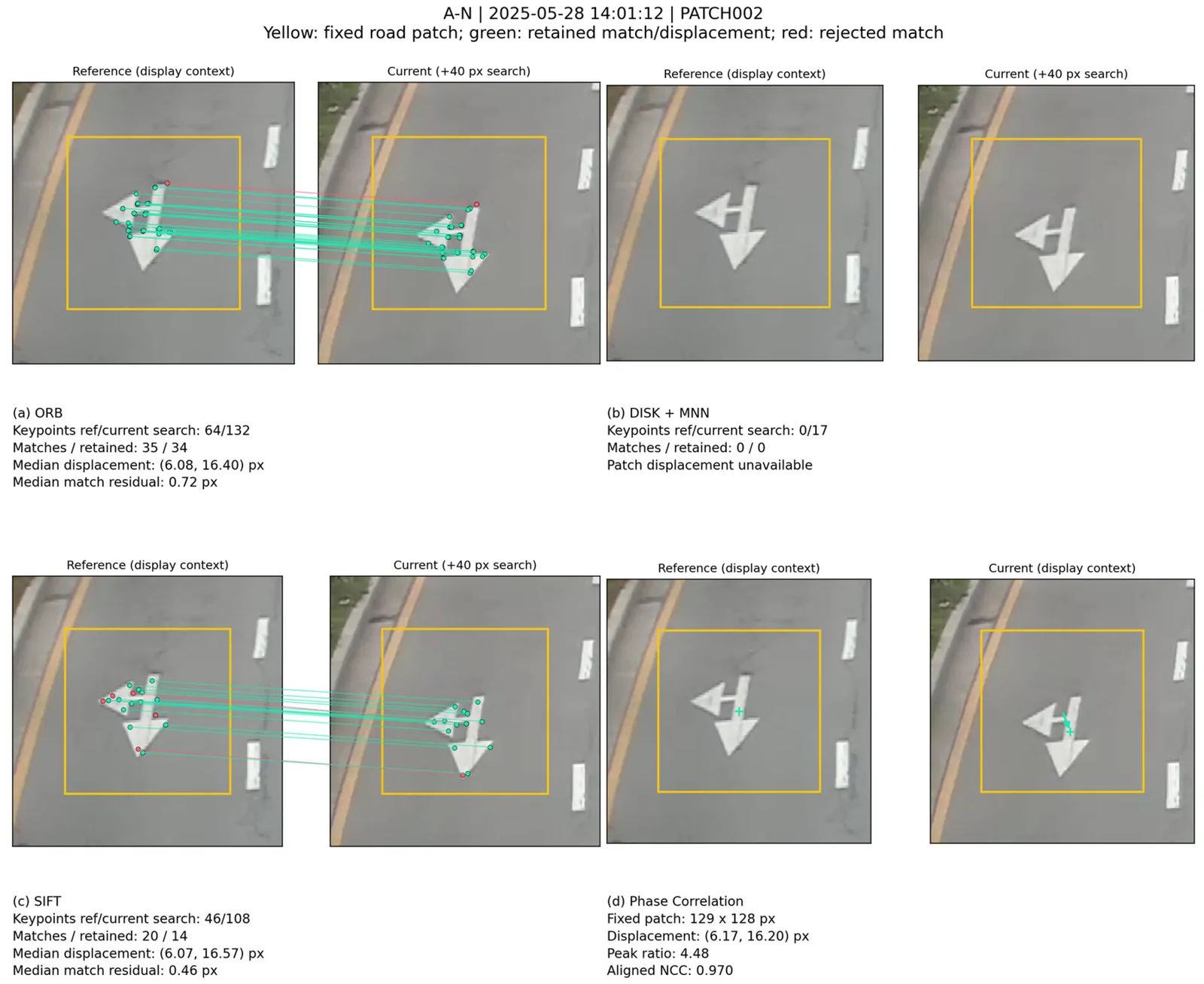
Protocol-matched registration on A-N, 28 May 2025 14:01:12, PATCH002. Yellow denotes the fixed patch; green and red denote retained and rejected sparse matches. For ORB, reference/current-search keypoints are 64/132, with 35 matches and 34 retained; for SIFT they are 46/108, with 20 matches and 14 retained. Globally extracted DISK has 0 reference keypoints in this patch and no matches. Phase displacement is (6.167, 16.199) px with aligned NCC 0.970. These are single-example diagnostics, not dataset-wide estimates.

Full-image-versus-patch comparisons retain the feature matching and homography settings but change feature support and, for classical methods, extraction scope. They are configuration comparisons. The patch-center affine protocol fixes a representation and transformation model, while direct-match controls test a less restrictive alternative. Figure 2 shows actual measurements for one common patch under the original protocol.

### 3.12. Same-Patch, Same-Affine Comparison of Registration Methods

Reference-image features for ORB, SIFT, DISK + MNN, and DISK + LightGlue were restricted to the same four road patches, with a 40 px search margin in the current image. Feature displacements within each patch were reduced to one representative displacement using the component-wise median and a median-absolute-deviation consistency test. To prevent a texture-rich patch from receiving a larger weight, every patch center provided exactly one equally weighted correspondence.

The patch-center protocol fits the same six-parameter affine model from at least three non-collinear patch centers, without homography fallback. Phase correlation also supplies one displacement per patch. This controls patch location, equal patch weighting and model, but does not establish equal information content or identical statistical efficiency for intensity registration and sparse descriptors. Direct individual-match comparisons are reported separately.

### 3.13. Evaluation Metrics for Mounting-Structure-Induced View Correction

Throughout, MHPE denotes the mean Euclidean horizontal position error, not the mean absolute error of separate coordinate components. Initial calibration distinguishes all-point in-sample error from held-out LOO error as defined in Section 3.7. Correction summaries first average the fixed visible landmarks in a frame, then weight frames equally. Cross-camera summaries explicitly identify camera-balanced means; paired views are grouped by installation site.

To prevent leakage from including an evaluation point in the initial homography estimate, *H*_0_,−*_i_^W^* was re-estimated after excluding every landmark i with a known ground coordinate. Let *C_t_,_m_* denote the current-to-reference transform estimated by method m for the current image. The predicted ground coordinate and horizontal error are

(7) $${\hat{q}}_{i,t,m}=\pi (H_{0},{{-}_{i}}^{W}\;C_{t,m}\;p_{i,t}),\;\;e_{i,t,m}\;=\;\|q_{i}\;-\;{\hat{q}}_{i,t,m}{\|}_{2}$$

Transform-estimation success was defined as producing a valid current-to-reference transform for a frame. For the global and patch-homography variants of ORB, SIFT, DISK + MNN, and DISK + LightGlue, success required valid feature descriptors, at least four matches, and a finite, non-degenerate homography with at least four inliers under USAC_MAGSAC with a 2 px threshold; the homography also had to be invertible. Insufficient features, matches, or inliers, as well as degenerate, singular, or numerically invalid homographies, were recorded as transform-estimation failures.

For feature methods in the same-patch, same-affine comparison, success required representative displacements from at least three patches with non-collinear centers, together with a finite, non-singular least-squares affine transform and inverse. The same three conditions were applied to the proposed Phase–Affine method. Patches outside the image boundary, an insufficient number of valid patches, collinear centers, affine degeneracy, and numerical errors were recorded as transform-estimation failures.

Patch gradient energy indicates whether sufficient texture is present through intensity variation and edges; the correlation peak ratio reflects displacement ambiguity through the separation between the largest and second-largest peaks; and post-registration normalized cross-correlation (NCC) measures appearance similarity between the reference and current patches after displacement correction. In the original 40-frame comparison, these three values were recorded only as diagnostic quantities for post hoc analysis of failure causes. They were not used to exclude patches or determine success; consequently, displacements marked as low-confidence were still included in affine estimation. In the separate extension, signed post-alignment NCC is explicitly used as the patch-selection variable; peak ratio and gradient energy remain diagnostic quantities.

Transform success is determined from image measurements and geometric checks, independently of GNSS error. All finite-frame MHPE values, including those at or above 10 m, enter the primary means reported in the original-frame correction results. Each table reports the finite-evaluation count and the count at or above 10 m against the original frame denominator. Missing transforms are not assigned zero error and are not successful identity updates. The 10 m cutoff is an offline diagnostic of large error, not an operational gate, physical safety limit or exclusion from the revised means. Secondary threshold counts at 0.5, 1, 2, 5 and 10 m are provided in Supplementary Materials S1 (S2_original40). For the 92-frame extension, a separate no-update policy uses the uncorrected error only on estimation/evaluation failures; this policy average is distinguished from correction-only finite-frame means.

### 3.14. Fixed-Center Patch-Size and NCC-Threshold Sensitivity

A separate experiment uses 23 approximately hourly images per camera (92 images), covering nominal hours 00:00–22:00. A-N, A-F, and B-N were recorded on 28 May 2025, whereas the replacement B-F sequence was recorded on 27 May 2025. Thus, these are four per-camera hourly sequences, not a synchronized same-date sample. Reference and current-image landmark coordinates were reviewed by the authors; landmarks not visible in a current image were omitted from that frame’s fixed evaluation set, without dropping frames based on correction performance. The extension uses 10, 22, 8, and 24 reference landmarks and 224, 487, 177, and 552 visible landmark–frame observations, respectively (1440 observations). This 64-reference-point temporal annotation snapshot is distinct from the 190-point, 14-camera static evaluation. The original 40-frame correction experiment retains its own fixed annotation snapshot.

The authors selected six persistent road-marking or road-texture centers per camera. Each center is held fixed while the square side length is varied over 50, 70, 100, 120, and 150 pixels at the original image resolution. Selection is manual, based on recognizable planar road structure and texture; this is not an automatic patch-proposal experiment. For each center and size, phase correlation produces a reference-to-current displacement. Signed post-alignment NCC is computed by rounding that displacement to integer pixels, taking the overlapping grayscale regions, subtracting each overlap mean, and dividing their inner product by the product of their Euclidean norms. The implementation assigns NCC zero when the overlap is smaller than four pixels in either dimension or the denominator is numerically negligible. Thus, NCC is an appearance-consistency score, not a calibrated probability of a correct displacement.

The baseline retains all successful finite phase displacements. The threshold sweep retains a patch only when its signed NCC is at least τ, with τ = 0.1, 0.2, …, 0.9. Absolute NCC is not used. Vehicle labels, GNSS errors, gradient energy, and correlation-peak ratios do not enter this selection rule. Phase measurements are cached so that all thresholds reuse identical displacement estimates. Each selected set is fitted with either equal-weight full affine least squares or a homography using OpenCV USAC_MAGSAC (2 px threshold, 10,000 iterations, confidence: 0.999; seed: 20,260,817). Affine requires at least three non-collinear centers; homography requires at least four correspondences and four inliers. Both fits check rank and numerical invertibility. The fitted reference-to-current mapping is inverted to obtain the current-to-reference correction used for evaluation. The model comparison also changes the estimator and therefore does not isolate transform degrees of freedom alone.

Each evaluation landmark uses a reference-to-ground homography fitted without that landmark. Current-image validation coordinates are never used to fit the correction. For each frame, the mean horizontal position error (MHPE) is the mean Euclidean east–north error over its fixed visible landmarks. Camera summaries weight the 23 frames equally. All finite errors, including errors ≥ 10 m, are retained. Estimation success here means that a transform and a finite evaluation are obtained; it does not imply an error below 10 m. If estimation or evaluation fails, the correction-only error remains missing and the failure is counted. A separate all-frame policy metric uses the uncorrected mapping on those failed frames only. This identity/no-update fallback is an explicit evaluation policy, not a successful correction, and it does not suppress large finite errors. Figures and summary tables report this all-frame policy metric together with the success count.

Vehicle occlusion is labeled by the authors at the 150 px setting and applied to the same center–frame observation at every size. Unannotated observations were confirmed clear. These labels indicate occlusion at the selected center rather than a size-specific fraction of covered pixels and are used only for post hoc interpretation. Across four cameras, 19 out of 552 center–frame observations per size are vehicle-occluded and 533 are clear; B-F contributes two occluded observations, both at C005, and 136 clear observations. The experiment contains 4 cameras × 23 frames × 5 sizes × 10 selection settings × 2 models = 9200 fits. Repeated sizes, thresholds, and frames are not treated as independent cameras. Per-camera minima are descriptive in-sample sensitivity results; no held-out dataset was used to choose a deployable threshold.

### 3.15. Landmark Count and Spatial Support Sensitivity

To test point-count and coverage sensitivity on the same reviewed 14 cameras, each landmark is withheld before training-set selection. We use 4, 6, 8, 10 and 12 training points when these are a strict subset of the remaining points, plus all n − 1 remaining points as ordinary LOO. For each strict-subset condition, ten draws use uniform sampling without replacement, compact nearest-neighbor selection around a random training anchor, or dispersed farthest-point selection from a random training anchor. Selection uses only training image locations, not held-out errors; draws may repeat the same subset. Both objectives receive exactly the same point IDs. The seed is 20,260,817, with camera-specific streams derived from source identifiers. We retain training hull area, withheld-point extrapolation distance and solver status. Image-region analysis uses image-y terciles of the fixed reviewed points; these are not surveyed camera ranges. Fixed-count comparisons of four and six points include all 14 cameras. Higher-count rows explicitly report fewer eligible cameras and must not be read as a same-camera count trend.

The same 14-camera dataset supports the geometry analysis; no separate camera cohort is introduced. We compare the mapping fitted from all points with each leave-one-out mapping on a fixed 41 × 41 image-coordinate bounding-box grid, retaining locations within the all-point convex hull. For an exterior LOO fold, this domain is partitioned into locations still inside the remaining-point hull and locations that lose convex-hull support after withholding the point. Both objectives use identical grid locations and folds. The grid samples image area, not equal ground area.

The Euclidean difference between the two predicted ground coordinates measures mapping sensitivity to removing a correspondence. It is not a ground-truth accuracy measurement at the grid locations. This diagnostic helps separate the loss of boundary support from held-out landmark error without discarding exterior folds or claiming that LOO is invalid. Interior and exterior results remain independently reported even where one objective performs worse. Repeated folds and paired cameras are not independent sites; these summaries are descriptive for seven installation sites.

Both coordinate sets undergo Hartley normalization to zero mean and root-mean-square radius √2. Normalized DLT initializes an eight-parameter homography with normalized h33 fixed to one. A custom Levenberg–Marquardt solver minimizes the squared target-coordinate residual, using at most 250 iterations, an initial damping of 0.001, a cost tolerance of 1 × 10^−14^ and a relative step tolerance of 1 × 10^−12^. Accepted decreases divide damping by three; unsuccessful candidates multiply it by ten, with up to 24 attempts per iteration. The pixel objective fits ground-to-image, then inverts; the ground objective fits image-to-ground. Isotropic target scaling preserves the objective optimum. Rank deficiency and non-finite projections are failures; finite estimates are retained even when the convergence flag is false, which is reported separately. The evaluation uses one fixed, author-reviewed point set for both objectives. All 28 all-point fits and all 380 LOO fits converged with finite predictions; no further points or folds were removed during these comparisons.

The count/layout experiment comprises 23,200 paired trials and 46,400 objective fits, including the 380 ordinary LOO fits. All predictions are finite. Twenty-nine pixel-objective fits in the compact six-point condition do not meet the convergence criterion and remain in the summaries with their flags. No subset is resampled or discarded because of its error. The ordinary LOO values reproduce the main 14-camera comparison within 10^−8^ m. Means first average draws and withheld points within a camera, then weight contributing cameras equally. Large finite errors remain in all means; medians of camera means and per-trial outputs are supplied for sensitivity to the tails.

### 3.16. Patch Number and Transformation Controls

Using the same approved six centers and cached phase measurements, we enumerate all 20 three-center, 15 four-center, six five-center and one six-center subsets at each of five sizes and each of 92 frames. The resulting 19,320 affine fits use no NCC selection, isolating prescribed support changes from a confidence threshold. Every subset is fixed across the sequence, rather than chosen to minimize each frame’s error. Per-subset and per-frame errors, hull coverage, failures, and all six leave-one-center-out cases are retained. A complementary analysis fits translation, similarity, affine and homography with equal-weight reference-to-current squared coordinate loss and no robust rejection. It contains 1840 size/frame/model evaluations; the fitted transform is explicitly inverted for current-to-reference evaluation. Homography uses nonlinear geometric refinement, while the other models have linear least-squares solutions. This comparison is separate from the earlier MAGSAC-homography sweep.

We also select one affine size/NCC configuration for each held-out camera using only the other three cameras, minimizing their equally weighted no-update-policy MHPE with deterministic tie-breaking. This is internal leave-one-camera-out configuration selection, not validation on independent sites. Non-overlap results use the fixed complement of all six patches at each size, keeping landmark membership common across subsets. Brightness is the mean grayscale intensity after excluding the top 125 rows; its correlations with NCC and error are descriptive and do not identify a causal illumination effect.

### 3.17. Direct Feature Fits and Extraction Diagnostics

For the original 40 frames, cached pre-aggregation ORB, SIFT, DISK + MNN and LightGlue correspondences are fitted directly to current-to-reference affine transforms with RANSAC: 2 px threshold, 5000 iterations, confidence 0.999, ten refinement iterations and at least three inliers. Three distinct patches are not required. This changes representation and robustness relative to patch-center least squares, so it is a strengthened baseline rather than a pure median-only ablation. DISK diagnostics retain the official weights and matcher while varying the full-image keypoint budget (2048/4096/8192 at a 1024 px longest edge), full-image native resolution, and independently extracted native-resolution patches with a shared 4096-keypoint cap. Current search rectangles have a 40 px margin. Five settings, two matchers and two affine representations produce 800 method–frame evaluations, with all finite errors retained.

A separate timing run benchmarks the direct-fit ORB/SIFT pipelines, native-crop DISK + MNN/LightGlue, and Phase–Affine on the same original 40-frame manifest. Reference descriptors or FFTs are cached once per camera. Three warm-ups per camera/method precede five timed repetitions per current frame. The frame statistic is the median repetition; the table reports the mean of those 40 medians. Synchronized core timing starts from a decoded image and includes current preprocessing, CPU–GPU transfer, extraction, matching and fitting; decoding, visualization and ground-error evaluation are excluded. CPU classical/phase and GPU learned methods run sequentially on the same RTX 3090 machine. All 80 learned method–frame errors reproduce the saved native-crop accuracy results within 1 × 10^−8^ m. These offline timings do not establish online throughput or transient response.

## 4. Results

### 4.1. All-Point Fitting and Interior/Exterior Leave-One-out Evaluation

Table 1 presents every camera on equal terms. Using the complete available control-point set, the ground objective reduces the camera-balanced all-point MHPE from 0.259 to 0.196 m (24.4%), with a lower MHPE for 10 out of 14 cameras. This is the principal fitted-calibration result: the surveyed control points are reproduced more accurately in horizontal coordinates on average. All-point RMSE decreases for all 14 cameras, and the square root of the camera-balanced mean squared error decreases from 0.394 to 0.257 m. This squared-error improvement is consistent with the ground objective, which minimizes squared metric residuals; it does not imply lower mean Euclidean error in every camera. Because these points also estimate the mapping, all-point results quantify fitting accuracy rather than independent prediction. Complementary held-out evaluation also shows lower camera-balanced error: interior LOO decreases from 0.331 to 0.318 m (4.1%), exterior LOO from 0.445 to 0.384 m (13.5%), and overall LOO from 0.398 to 0.348 m (12.6%). Seven out of 14 cameras improve in overall LOO. The later summary of evaluation protocols therefore separates the all-point fitting benefit from the geometry-dependent predictive benefit.

### 4.2. Sensitivity to Perturbed Image Correspondences

Across the same 14 cameras, the matched 1–2 px perturbation experiment decreases the camera-balanced mean LOO MHPE from 0.409 to 0.381 m, a 7.0% reduction. The ground objective yields a lower camera-balanced mean in 960 out of 1000 repetitions. However, the trial-averaged error improves in only seven out of 14 cameras, as shown in Table 2. All 380,000 objective fits converge and give finite predictions.

These trials characterize sensitivity to the specified synthetic image-point perturbations while keeping each validation image location and its GNSS coordinate fixed. They do not establish that human marking errors follow this distribution, and 1000 repetitions do not constitute 1000 independent deployments. The camera-wise variability remains important even when the pooled comparison favors the ground objective.

### 4.3. Camera-Wise Full-Image and Patch Correction Results

Table 3, Table 4, Table 5 and Table 6 report each camera’s original ten frames without error truncation. Each entry gives finite evaluations out of ten, MHPEs over every finite frame, and the number of finite frames with an MHPE of at least 10 m. Thus, both missing estimates and large errors remain visible.

The no-correction MHPE values are 3.000, 2.156, 0.381 and 3.349 m for A-N, A-F, B-N and B-F. Phase–Affine has ten finite evaluations per camera, with MHPEs of 0.415, 0.340, 0.348 and 0.466 m, respectively; no frame reaches 10 m.

Over the original 40 frames, patch–homography ORB, SIFT, DISK + MNN and DISK + LightGlue produce 36, 37, 40 and 38 finite evaluations. Their untruncated means are 16.141, 4.105, 40.351 and 26.256 m; 11, 3, 13 and 17 frames reach 10 m, respectively. These outcomes differ substantially from averages conditioned on errors below 10 m.

The large-error tails demonstrate instability in some narrow-support configurations. They do not establish an intrinsic limitation of the feature extractors or matchers: feature allocation, match geometry and transformation representation change the outcome. Section 4.10 evaluates direct individual matches and alternative DISK extraction settings without discarding these large errors.

### 4.4. Same-Patch, Same-Affine Registration Results

Under equal-weight patch-center affine fitting, ORB, SIFT, DISK + MNN and DISK + LightGlue produce 24, 36, 20 and nine finite evaluations. Their untruncated means are 26.109, 41.831, 5.203 and 6.826 m, including four, 10, five and three frames at or above 10 m. Phase–Affine produces 40/40 finite evaluations with 0.392 m. Table 7 includes all such errors rather than conditioning the comparison on successful accuracy.

In the threshold-sensitivity analysis, Phase–Affine remained valid for 40/40 frames at every evaluated threshold of 0.5, 1, 2, 5, and 10 m. At the 0.5 m threshold, the valid-frame counts were 15 for ORB, 22 for SIFT, 11 for DISK + MNN, and five for DISK + LightGlue. The 40/40 result of the proposed method was therefore not an artifact of using the 10 m criterion.

The original patch-center representation is particularly restrictive when a matcher supplies correspondences from only one or two patches. Direct affine estimation can use individual matches without requiring three distinct patch centers. The stronger controls in Section 4.10 therefore constrain interpretation of Table 7: its low learned-method availability is configuration-specific, not a general ranking of matching algorithms.

As a secondary common-support comparison, all five original patch-center affine methods have finite transforms and errors on only four frames, all from A-N. On exactly those frames, ORB, SIFT, DISK + MNN, DISK + LightGlue and Phase–Affine yield 0.580, 0.406, 0.432, 2.577 and 0.416 m, respectively. No 10 m filter is used to form this intersection. Because three cameras contribute no common frame, this selected comparison is not a representative four-camera ranking and does not replace the failure-inclusive counts and means. The stronger native-patch learned baselines and Phase–Affine can instead be compared over all 40 frames.

### 4.5. Computational Cost

As summarized in Table 8, the mean fixed-reference current-frame core runtimes under the same-patch, same-affine protocol were 10.38 ms for ORB, 16.73 ms for Phase–Affine, 50.31 ms for SIFT, 86.61 ms for DISK + MNN, and 150.37 ms for DISK + LightGlue. Phase–Affine was 1.61 times slower than ORB, but it was the only method that produced a transform for all 40 frames. It was 3.01, 5.18, and 8.99 times faster than SIFT, DISK + MNN, and DISK + LightGlue, respectively. Thus, the result does not imply that phase correlation is always the fastest method; rather, in the present dataset and implementation, it combined the second-shortest runtime with the highest transform-estimation rate.

The accuracy and runtime experiments used the same 40-frame manifest. Transform-success states across all 200 frame–method combinations and the match counts in successful cases were consistent between the two experiments.

### 4.6. Patch-Size, NCC-Threshold, and Vehicle-Occlusion Sensitivity

Table 9 summarizes the hourly sensitivity dataset. The sensitivity analysis shows that increasing the NCC threshold does not monotonically improve ground-position accuracy. Restricting support can remove unreliable displacements, but it can also leave too few or poorly distributed centers. Table 10, Table 11, Table 12 and Table 13 summarize each size using its lowest observed all-frame error across the sweep, alongside the all-patch result at the same size. The selected threshold is reported to make this evaluation-set selection explicit. These are sensitivity summaries rather than externally validated optimal settings. Appendix A (Table A1, Table A2, Table A3, Table A4, Table A5, Table A6, Table A7 and Table A8) reports every patch size and NCC threshold, together with the all-frame error and estimation-success count.

The lowest observed affine errors were 0.446 m for A-N (120 px, τ = 0.6), 0.180 m for A-F (150 px, τ = 0.4), 0.306 m for B-N (100 px, τ = 0.5), and 0.793 m for B-F (100 px, τ = 0.4), each with 23/23 successful estimations. The homography minima were 0.455, 0.228, 0.314, and 0.805 m, respectively, also with 23/23 success. For B-F, the homography minimum occurred at 120 px and τ = 0.2. These are in-sample descriptive minima, not independently validated operating settings or evidence for a universal model or threshold advantage.

Failure-aware reporting materially changes the interpretation of strict thresholds. For A-F with 70 px patches and homography at τ = 0.9, only 13/23 frames produced finite evaluations; four of those exceeded 10 m. The successful-frame mean was 282.086 m, and the maximum was 2405.388 m. Including uncorrected fallback on the ten failed frames yielded an all-frame mean of 160.473 m. The extreme finite errors were retained. Even Affine was not immune: at 150 px on A-F, increasing τ from 0.4 to 0.5 increased the all-frame mean from 0.180 to 1.873 m. Therefore, high appearance similarity alone is insufficient to ensure a stable geometric correction; the individual contributions of center geometry and displacement error require further analysis.

Table 14 summarizes the pooled NCC statistics for vehicle-occluded and clear observations.

**Figure 3:**
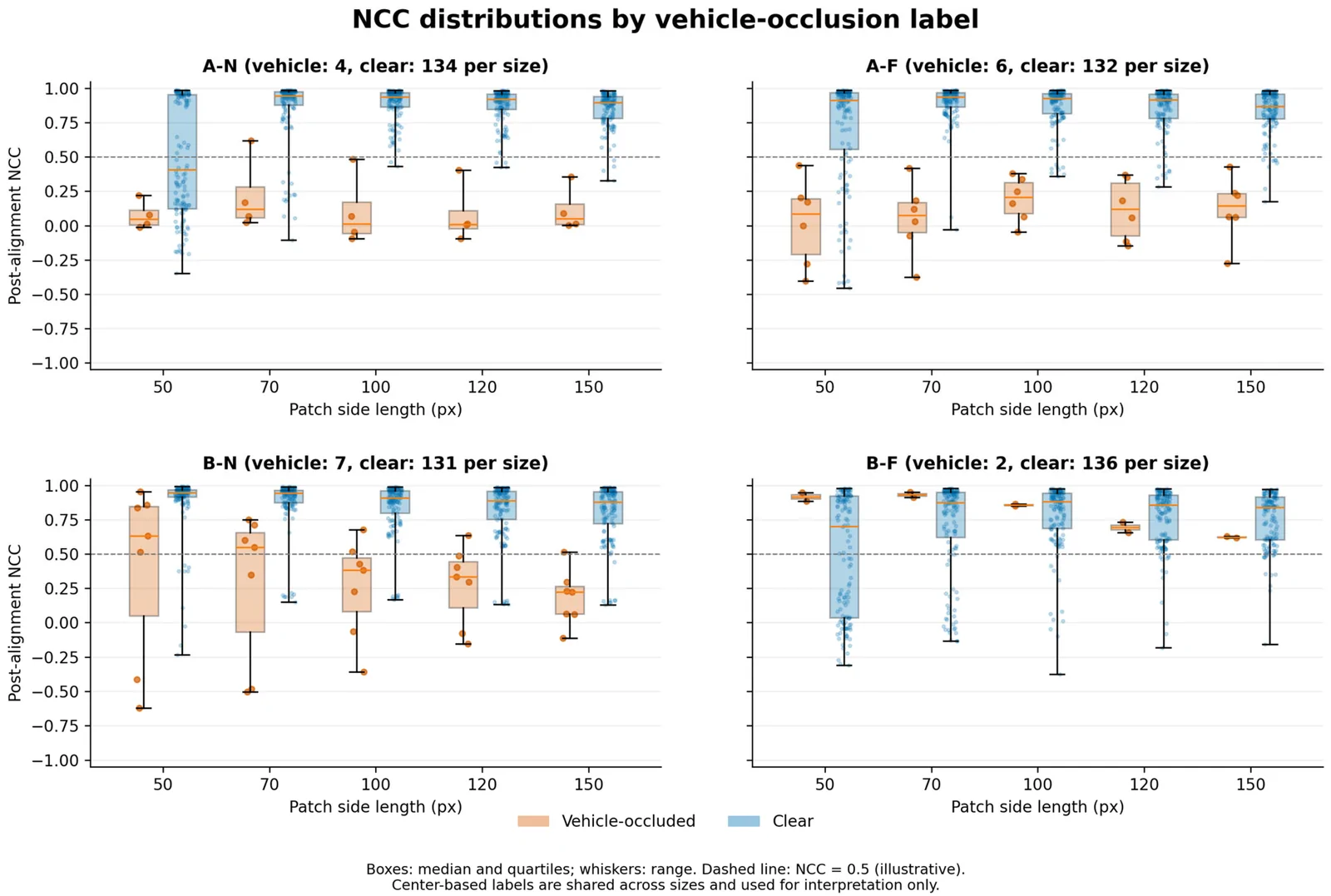
Signed post-alignment NCC distributions for vehicle-occluded and clear center–frame observations, separated by camera. Boxes show medians and quartiles; whiskers span the observed ranges. The dashed 0.5 line is illustrative, not a validated operating threshold. Labels are shared across sizes and are not used in patch selection. B-F has only two vehicle-labeled observations per size; its occlusion distribution should be interpreted cautiously.

Figure 3 shows the camera-specific NCC distributions. Vehicle-occluded observations had pooled mean NCC values between 0.196 and 0.268, compared with 0.647–0.845 for clear observations across the five sizes. At the illustrative threshold 0.5, 12–16 out of 19 vehicle-occluded observations were rejected per size, while clear observations were also rejected: 172/533 at 50 px and 28–50/533 at 70–150 px. In particular, the two vehicle-labeled B-F observations were retained at this threshold across all five sizes. A vehicle label therefore does not guarantee low NCC, and NCC is not a complete occlusion detector. The pooled association should not be interpreted as a causal explanation of every error. Displacement beyond patch support is another plausible mechanism but was not independently labeled and is not asserted as an established dominant cause.

### 4.7. Full-Image Feature Baselines on the Same Hourly Sequences

Table 15, Table 16, Table 17 and Table 18 compare uncorrected mapping, full-image ORB, SIFT, DISK + MNN, and DISK + LightGlue homography correction, and patch phase correction on each camera’s same 23 frames and visible landmarks. The patch rows use the common 150 px size with all finite patch displacements and no NCC threshold, rather than selecting a per-camera minimum. The learned methods use the official DISK extractor and LightGlue matcher; the full-image field excludes the timestamp overlay. All finite errors are retained. Estimation counts and counts with an MHPE ≥ 10 m are separated; the latter are not excluded from means. Identity fallback applies only to failed estimation/evaluation. These are scope-and-model pipeline comparisons, not a controlled isolation of the matching algorithm, and no new matched-runtime claim is made.

Full-image feature matching remained competitive: DISK + LightGlue produced 0.420, 0.172, 0.313, and 0.791 m on A-N, A-F, B-N, and B-F. All four feature pipelines yielded finite evaluations in all 23 frames per camera, but ORB on B-F had two errors ≥ 10 m and an all-frame mean of 3.712 m, exceeding the uncorrected 3.545 m. A finite transform is therefore not synonymous with accurate correction. At the common unfiltered 150 px setting, Phase–Affine errors were 0.619, 0.519, 0.442, and 0.883 m. NCC selection reduced these errors at the descriptive settings in Table 10, Table 11, Table 12 and Table 13, but this in-sample selection must not be presented as a tuned-versus-untuned proof of superiority over full-image methods.

### 4.8. Interpretation of Interior and Exterior LOO

**Figure 4:**
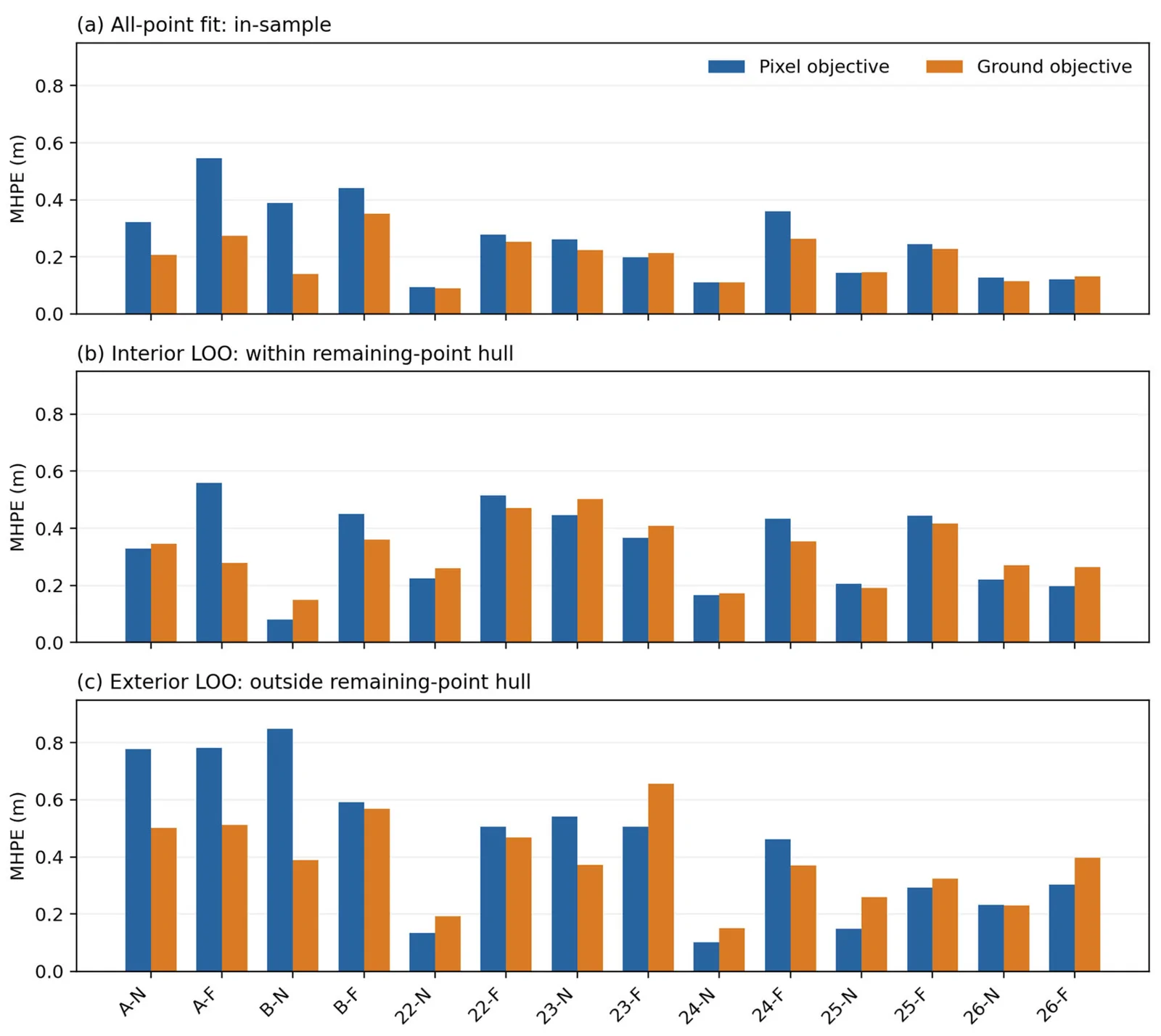
Pixel and ground objectives on all 14 cameras: (**a**) all-point in-sample fitting, (**b**) interior LOO and (**c**) exterior LOO. Axes share the same linear error scale. Cameras are not separated into original and additional cohorts; every camera has equal weight in the aggregate summaries.

Table 19 and Figure 4 summarize the complete 14-camera comparison without dividing cameras by acquisition stage. Exterior LOO has a higher camera-balanced error than interior LOO for both objectives. Withholding a boundary landmark can shrink the training hull and turn evaluation at that location into extrapolation. Interior LOO instead evaluates an unseen point within the remaining support. These are different operating conditions, so presenting only their pooled mean would conceal relevant geometry.

Removing exterior landmarks changes the fitted mapping beyond the withheld location. Mean ground-coordinate mapping changes within the retained hull are 0.035/0.032 m (pixel/ground), compared with 0.114/0.120 m in the region that loses hull support. These grid differences measure sensitivity, not accuracy. The ground objective therefore does not uniformly stabilize unsupported regions, even though its exterior-LOO held-out error is lower on average.

A concrete boundary case is landmark p001 in 22-F: withholding it removes approximately 28.5% of the image hull area, and the maximum grid mapping change reaches approximately 1.064 m for the pixel objective and 1.059 m for the ground objective. This illustrates why retaining spatially distributed boundary landmarks matters. It does not justify deleting the fold or classifying its error as an algorithm-independent failure.

Together, the three evaluations support improved horizontal calibration fit with a bounded predictive claim. All-point fitting assesses the mapping obtained from the complete available control-point set and shows how accurately those surveyed points are reproduced. Interior LOO additionally tests unseen points within the remaining support, while exterior LOO tests extrapolation and sensitivity to boundary-point removal. Their average improvements provide complementary evidence beyond fitting, although the modest interior gain and mixed camera-wise results do not establish uniform predictive superiority. These limitations do not negate the observed all-point improvement; they identify where that improvement can and cannot be extended to unseen locations. The following count/layout experiment uses the same reviewed coordinates to examine this dependence.

**Figure 5:**
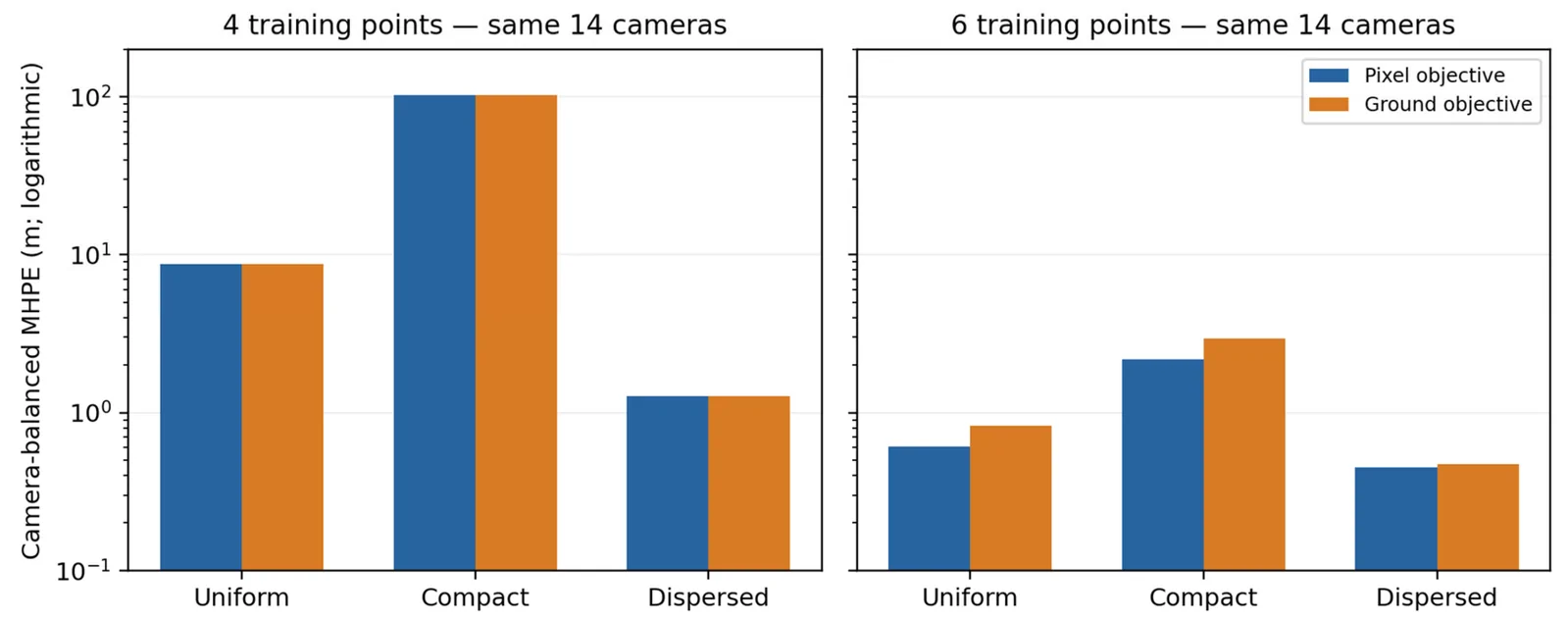
Count/layout sensitivity for four and six training points using the same 14 cameras. The logarithmic error axis retains extreme finite projections. Each bar is the equal-camera mean, not the mean over independent deployments.

Table 20 and Figure 5 show that support geometry can matter more than the choice of objective. With four points, a non-degenerate homography can interpolate the training correspondences, so the two objectives produce essentially the same mapping. Uniform four-point sampling gives 8.735 m for both objectives, compact sampling gives 102.373 m, and dispersed sampling gives 1.271 m. The respective medians of camera means are 4.579, 17.346 and 0.858 m. These large finite errors are retained and demonstrate extrapolation sensitivity, not a claim that all calibrations have such errors.

With six training points and the same 14 cameras, uniform sampling yields 0.613/0.828 m (pixel/ground), compact sampling 2.164/2.950 m and dispersed sampling 0.448/0.474 m. Their mean image hull fractions are 0.144, 0.077 and 0.217. The ground objective is worse in all three six-point aggregates, despite its lower ordinary LOO mean with all remaining points. Thus, optimizing metric residuals does not compensate for sparse or concentrated support. Higher-count comparisons use different eligible camera sets and are not evidence of a monotonic camera-independent trend.

At counts 8, 10 and 12, the strict-subset comparison includes 11, seven and five cameras, respectively. If a requested count equals all remaining points, that camera contributes to ordinary LOO rather than a repeated layout condition. There are no missing numerical evaluations; 29 out of 1900 compact six-point pixel fits are finite but not converged. Their errors are included. Complete per-camera means, medians, maxima, sample IDs and convergence flags are provided in Supplementary Materials S1 (S1_static14).

Table 21 separates ordinary LOO by image-y tercile. Upper-image landmarks yield 0.567/0.404 m, middle landmarks 0.363/0.357 m, and lower landmarks 0.270/0.292 m. The ground objective therefore reduces the upper-region error but slightly increases the lower-region error. Image position is only a descriptive proxy for projection sensitivity and is not a measured camera-to-landmark distance. These region results do not establish an intrinsic near/far camera effect independent of layout.

### 4.9. Independent Patch Counts and Model Choice

**Figure 6:**
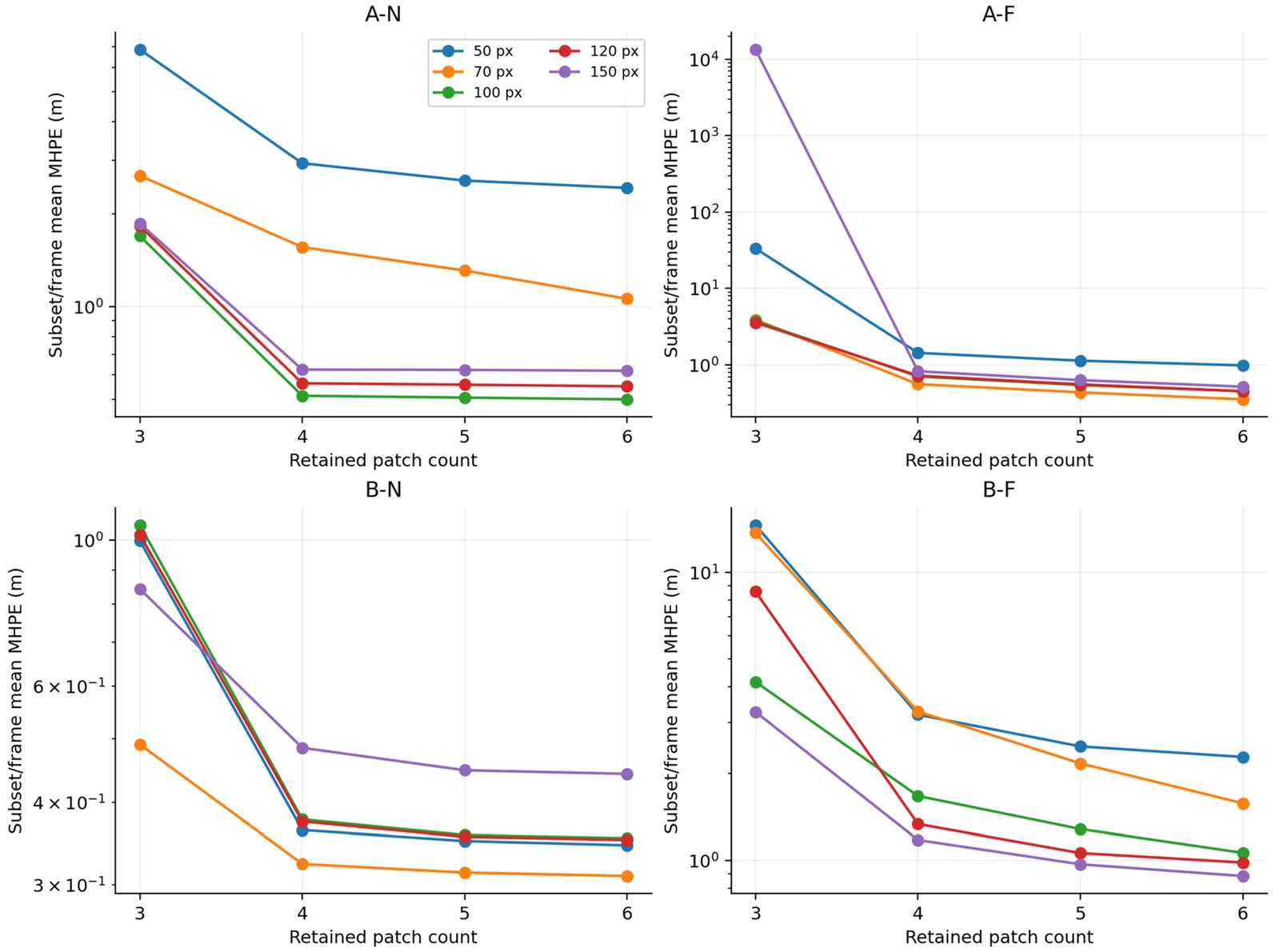
Affine error across all fixed-center subsets. Each curve uses one patch size; the log scale retains unstable configurations. The means are sensitivity summaries, not errors from independent additional frames.

Figure 6 summarizes every prescribed subset without selecting the best subset. At 150 px (Table 22), three-patch fits can be unstable despite being numerically estimable. A-F is especially sensitive, with a mean over subsets and frames of 13,435.702 m, driven by a highly unstable configuration; this value is retained. Four to six patches reduce that mean, but improvement is not monotonic in every scene or size. Leave-one-center-out and hull-association tables expose which approved configurations supply useful support without asserting robustness to arbitrary unseen layouts.

Table 23 compares equal-weight, non-robust transformation models using all six patch centers.

Translation yields lower error than unfiltered affine on A-N and A-F, while similarity is lower on B-N and B-F. Unfiltered least-squares homography reaches 9.164 m on A-F, unlike the MAGSAC-homography result in the earlier sweep. Thus, neither affine nor additional projective freedom is uniformly optimal. Under the leave-one-camera-out configuration selection, the held-out MHPE values are 0.448, 0.187, 0.309 and 0.798 m for A-N/A-F/B-N/B-F, all with 23/23 finite evaluations. The selected size/threshold pairs are 150/0.3, 100/0.5, 100/0.4 and 150/0.4, respectively. These results reduce same-camera tuning but do not establish independent-site generalization.

Brightness–mean-NCC Spearman correlations are 0.548, 0.698, 0.085 and 0.625 for A-N/A-F/B-N/B-F; brightness–error correlations are −0.407, −0.385, −0.410 and 0.186. Different signs for error and the weak B-N NCC association preclude a single causal explanation based on darkness. Vehicle-center labels, texture diagnostics and geometric support should be interpreted jointly; NCC is not an occlusion detector. Supplementary Materials S1 (S3_hourly92) includes fixed non-overlap landmark results, all center coordinates, subset hull coverage and per-patch gradient energy.

With all six 150 px patches and no NCC gate, evaluation restricted to visible landmarks outside the entire patch union yields affine MHPEs of 0.717, 0.588, 0.488 and 0.900 m for A-N/A-F/B-N/B-F, with 23 finite-frame evaluations each. The complement is fixed across patch subsets at each size. This avoids evaluating inside the measurement regions, although it changes the landmark set and is not a causal overlap-effect comparison.

### 4.10. Stronger Feature Baselines and Failure Diagnosis

Table 24 shows that Phase–Affine has a lower observed MHPE under the present annotations on the original 40 frames, even against strengthened patch-based learned-feature baselines. It achieves 0.392 m over 40/40 finite evaluations, compared with 0.462 m for native-resolution patch DISK + LightGlue with direct robust affine fitting and 0.554 m for DISK + MNN, both also finite on 40/40 frames. Direct fitting raises LightGlue availability from 9/40 under patch-center aggregation to 39/40 with globally extracted patch features, and native-resolution crops raise it to 40/40. Thus, the original 6/40 accuracy-qualified result reflects a restricted pipeline and does not establish that LightGlue is intrinsically unsuitable for road patches. The 0.070 m difference between Phase–Affine and native-resolution patch DISK + LightGlue is descriptive: it does not establish an accuracy advantage beyond annotation uncertainty because no formal repeatability study was performed. This result concerns the 40-frame patch-based comparison; the hourly full-image comparisons are interpreted separately.

The recorded support explains much of the original LightGlue availability loss: six out of 16 original reference patches contain no DISK keypoints under global extraction. Thirty LightGlue frames fail for insufficient supported patches, despite having 5–20 matches overall; 25 support two patches and five support one. This is an observed support mechanism, not a diagnosis that every error is caused by feature scarcity. All five extraction settings, both affine representations, counts, inliers, errors and failure reasons are provided with the analysis data. Figure 2 adds quantitative keypoint, match, retained-match and displacement measurements for the exact illustrated example.

### 4.11. Runtime of the Strengthened Baselines

Table 25 establishes the measured computation advantage of Phase–Affine in the strengthened patch-based comparison: its mean core time is 9.858 ms, compared with 106.094 ms for native-resolution patch DISK + LightGlue with direct affine fitting. Together with the 0.392 versus 0.462 m MHPE and 40/40 finite evaluations for both methods, these results show lower observed MHPEs under the present annotations and a clearer measured runtime advantage under this protocol and hardware environment. The small accuracy difference is not claimed to exceed annotation uncertainty. ORB remains faster but has large finite errors. Table 8 describes the original ten-repeat patch-center benchmark; Table 25 is a separate five-repeat direct-fit benchmark, and its times are not spliced into the older run. This timing result is not transferred to the 92-frame experiment or interpreted as an end-to-end frame-rate guarantee.

## 5. Discussion

The ground objective improves fitted calibration: all-point MHPE decreases by 24.4% across the equally weighted 14 cameras, and all-point RMSE decreases for every camera. These are gains at the surveyed control points, not independent predictions. Interior and exterior LOO also show lower mean errors at unseen points, but improvement in seven out of 14 cameras and the smaller interior gain limit claims of uniform predictive benefit. The matched six-point experiment shows that the loss cannot compensate for restricted geometric support. In correction, Phase–Affine has a lower observed mean error under the present annotations and lower measured core runtime than native-resolution patch DISK + LightGlue with direct affine fitting on the original 40 frames. Strengthening the learned-feature pipeline resolves much of its earlier availability loss. On the hourly sequences, full-image learned features are more accurate than unfiltered 150 px Phase–Affine. Patch size, selection and model complexity therefore remain material conditions of performance. Simpler transformations outperform affine in some matched settings, and patch count or NCC alone does not guarantee reliable updates. The evidence supports a lightweight road-patch correction option, not a universally optimal matcher or transformation model.

Static evaluation covers 14 cameras at seven sites, whereas temporal correction remains restricted to four cameras at two sites, with one 23-frame sequence per camera on two adjacent dates. The 14-camera static dataset therefore does not establish temporal robustness at all seven sites. Neither season/weather generalization nor long-term surface degradation is established. Patch-subset tests use the approved six centers and cannot establish robustness to arbitrary new layouts. Texture and brightness analyses are observational, with geometry, illumination and occlusion potentially confounded. Leave-one-camera-out setting selection is internal validation, not an independent-site deployment study, because paired views share sites. Manual center selection remains necessary. Evaluation is limited to points on the selected approximately planar road sections with an approximately constant grade. This geometric selection is based on the authors’ assessment of the road sections; point elevations, slope angles and deviations from planarity were not surveyed for this study. The results therefore do not establish performance as a function of grade or generalize to changing-grade or non-planar roads. No full 3D or 6-DoF accuracy is claimed. Phase correlation assumes local translation; subsequent affine fitting does not remove failures caused by within-patch rotation, scale, perspective or occlusion. No implemented object detector or validated safety gate is claimed.

## 6. Conclusions

This study demonstrates improved horizontal-coordinate calibration fit and efficient road-patch phase correction for points on selected approximately planar road sections with an approximately constant grade. Using the complete available control-point sets from 14 equally weighted cameras, the ground objective reduces all-point MHPE from 0.259 to 0.196 m (24.4%); MHPE improves for 10 cameras and all-point RMSE for all 14. This is an in-sample improvement at the same surveyed control points used to estimate the mapping, not an independent predictive result. Complementary interior LOO decreases from 0.331 to 0.318 m and exterior LOO from 0.445 to 0.384 m. Overall LOO decreases from 0.398 to 0.348 m, with improvement in seven out of 14 cameras, so predictive benefit depends on point geometry. The 1–2 px perturbation study yields a 7.0% camera-balanced mean reduction at an author-motivated scale rather than a measured annotation-error distribution. On the original 40 correction frames, Phase–Affine achieves 0.392 m and a 9.858 ms mean core time, compared with 0.462 m and 106.094 ms for native-resolution patch DISK + LightGlue with direct affine fitting, both with 40/40 finite evaluations. The 0.392 versus 0.462 m comparison is a lower observed MHPE under the present single-annotator annotations; without formal repeatability measurements, it does not establish that this small difference exceeds annotation uncertainty. The measured runtime advantage is clearer under the stated benchmark conditions. The 92-frame analysis retains all finite large errors and counts missing estimates while identifying sensitivity to patch size, count, layout, NCC selection and model complexity. Full-image learned features perform better than the unfiltered 150 px affine configuration, and the ground objective does not outperform the pixel objective in the common six-point aggregates. The demonstrated advantages therefore concern complete-set calibration fit and a lower measured runtime with a lower observed MHPE in the evaluated patch-based comparison, with generalization constrained by geometric support and registration conditions.

For deployment, calibration geometry, patch support and update reliability require scene-specific verification. Further work should characterize GNSS horizontal measurement uncertainty, independently validate configuration selection, and evaluate additional temporal environments. Automatic patch proposals, non-planar road models and downstream object-ground-contact localization remain outside the demonstrated scope.

## Figures and Tables

**Table 1:** Camera-wise horizontal calibration errors for all 14 cameras. Each error entry is pixel/ground MHPE (m). Counts are all/interior/exterior points. All-point evaluation is in-sample; both LOO categories withhold the evaluated landmark.

Camera	Counts	All-Point (m)	Interior LOO (m)	Exterior LOO (m)
A-N	10/6/4	0.321/0.206	0.328/0.346	0.777/0.503
A-F	26/19/7	0.545/0.274	0.559/0.278	0.782/0.512
B-N	8/1/7	0.388/0.140	0.081/0.149	0.850/0.388
B-F	24/14/10	0.441/0.351	0.451/0.361	0.592/0.569
22-N	10/2/8	0.093/0.090	0.225/0.260	0.133/0.191
22-F	12/6/6	0.278/0.253	0.515/0.472	0.505/0.467
23-N	10/4/6	0.262/0.222	0.446/0.503	0.542/0.371
23-F	9/4/5	0.198/0.212	0.367/0.408	0.507/0.657
24-N	15/9/6	0.109/0.111	0.165/0.172	0.101/0.150
24-F	18/11/7	0.360/0.264	0.434/0.355	0.462/0.369
25-N	16/8/8	0.143/0.145	0.206/0.190	0.147/0.258
25-F	13/4/9	0.244/0.228	0.444/0.417	0.293/0.323
26-N	10/4/6	0.127/0.116	0.221/0.271	0.231/0.229
26-F	9/5/4	0.120/0.132	0.197/0.265	0.303/0.397
Equal-camera mean	190/97/93	0.259/0.196	0.331/0.318	0.445/0.384

**Table 2:** All 14 cameras: mean LOO horizontal error over 1000 matched 1–2 px perturbation trials. Improvement is calculated from the two mean errors; negative values indicate worse ground-objective error.

Camera	Pixel/Ground MHPE (m)	Relative Reduction
A-N	0.516/0.468	9.4%
A-F	0.622/0.360	42.2%
B-N	0.757/0.429	43.3%
B-F	0.514/0.470	8.5%
22-N	0.176/0.238	−35.1%
22-F	0.527/0.504	4.4%
23-N	0.538/0.487	9.5%
23-F	0.448/0.564	−25.7%
24-N	0.147/0.177	−20.5%
24-F	0.449/0.396	11.8%
25-N	0.186/0.237	−27.5%
25-F	0.357/0.380	−6.5%
26-N	0.231/0.264	−14.6%
26-F	0.261/0.353	−35.2%
Equal-camera mean	0.409/0.381	7.0%

**Table 3:** A-N: original ten-frame full-image and patch comparison without error truncation.

Method	Full: Finite/MHPE/≥10 m	Patch: Finite/MHPE/≥10 m
No correction	10/10/3.000/0	–
ORB	10/10/0.410/0	10/10/0.709/0
SIFT	10/10/0.403/0	10/10/0.449/0
DISK + MNN	10/10/0.402/0	10/10/0.721/0
DISK + LightGlue	10/10/0.404/0	10/10/2.136/0
Proposed Phase–Affine	–	10/10/0.415/0

**Table 4:** A-F: original ten-frame full-image and patch comparison without error truncation.

Method	Full: Finite/MHPE/≥10 m	Patch: Finite/MHPE/≥10 m
No correction	10/10/2.156/0	–
ORB	10/10/0.386/0	10/10/21.268/3
SIFT	10/10/0.346/0	10/10/0.573/0
DISK + MNN	10/10/0.361/0	10/10/65.556/3
DISK + LightGlue	10/10/0.356/0	10/10/24.304/4
Proposed Phase–Affine	–	10/10/0.340/0

**Table 5:** B-N: original ten-frame full-image and patch comparison without error truncation.

Method	Full: Finite/MHPE/≥10 m	Patch: Finite/MHPE/≥10 m
No correction	10/10/0.381/0	–
ORB	10/10/0.354/0	9/10/20.551/5
SIFT	10/10/0.353/0	9/10/14.971/3
DISK + MNN	10/10/0.352/0	10/10/4.943/2
DISK + LightGlue	10/10/0.355/0	9/10/27.749/5
Proposed Phase–Affine	–	10/10/0.348/0

**Table 6:** B-F: original ten-frame full-image and patch comparison without error truncation.

Method	Full: Finite/MHPE/≥10 m	Patch: Finite/MHPE/≥10 m
No correction	10/10/3.349/0	–
ORB	10/10/0.624/0	7/10/25.193/3
SIFT	10/10/0.491/0	8/10/0.866/0
DISK + MNN	10/10/0.481/0	10/10/90.182/8
DISK + LightGlue	10/10/0.478/0	9/10/53.733/8
Proposed Phase–Affine	–	10/10/0.466/0

Note for Table 3, Table 4, Table 5 and Table 6: Entries are finite count/MHPE (m)/count ≥ 10 m. Missing estimates remain in the denominator but have no correction error. No correction uses the identity baseline. Learned patch–homography rows use globally extracted features restricted to patches; native-resolution crop controls are reported in the strengthened-baseline results section.

**Table 7:** Original equal-weight patch-center affine protocol. Entries are finite count/untruncated MHPE (m)/count ≥ 10 m.

Method	A-N	A-F	B-N	B-F	Overall
ORB–Affine	10/10/8.920/1	10/10/53.563/3	2/10/0.333/0	2/10/0.555/0	24/40/26.109/4
SIFT–Affine	10/10/36.507/2	10/10/27.240/2	7/10/23.819/2	9/10/77.966/4	36/40/41.831/10
DISK + MNN–Affine	10/10/0.483/0	0/10/–/0	10/10/9.924/5	0/10/–/0	20/40/5.203/5
DISK + LightGlue–Affine	4/10/2.577/0	0/10/–/0	5/10/10.226/3	0/10/–/0	9/40/6.826/3
Phase–Affine	10/10/0.415/0	10/10/0.340/0	10/10/0.348/0	10/10/0.466/0	40/40/0.392/0

**Table 8:** Original patch-center benchmark: fixed-reference current-frame core runtime using one displacement per patch and equal-weight affine fitting. Means of per-frame median times from ten repetitions after five warm-ups. This is a separate pipeline and timing run from the direct-fit benchmark reported in the strengthened-baseline results section; absolute times must not be compared across the two benchmarks.

Method	Transforms Estimated	Mean Core Time (ms)
ORB–Affine	24/40	10.38
SIFT–Affine	36/40	50.31
DISK + MNN–Affine	20/40	86.61
DISK + LightGlue–Affine	9/40	150.37
Phase–Affine	40/40	16.73

**Table 9:** Separate hourly sensitivity dataset. A-N, A-F, and B-N: 28 May 2025; B-F: 27 May 2025. Each camera contributes 23 frames.

Camera	Frames	Reference Points	Visible Point–Frames	Uncorrected MHPE (m)
A-N	23	10	224	3.113
A-F	23	22	487	2.361
B-N	23	8	177	0.457
B-F	23	24	552	3.545

**Table 10:** A-N: all-patch error and descriptive minimum across NCC settings at each size. Errors are all-frame MHPE (m); success has no ground-error cutoff.

Size (px)	Model	All Patches (m)	Selected τ	Minimum (m)	Success
50	Affine	2.433	0.9	2.248	10/23
50	Homography	5.592	0.9	3.186	5/23
70	Affine	1.061	0.6	0.449	23/23
70	Homography	0.988	0.6	0.569	23/23
100	Affine	0.499	0.7	0.446	23/23
100	Homography	0.463	0.4	0.462	23/23
120	Affine	0.551	0.6	0.446	23/23
120	Homography	0.458	all	0.458	23/23
150	Affine	0.619	0.6	0.447	23/23
150	Homography	0.455	0.3	0.455	23/23

**Table 11:** A-F: all-patch error and descriptive minimum across NCC settings at each size. Errors are all-frame MHPE (m); success has no ground-error cutoff.

Size (px)	Model	All Patches (m)	Selected τ	Minimum (m)	Success
50	Affine	0.985	0.6	0.554	21/23
50	Homography	5.139	0.3	1.449	19/23
70	Affine	0.353	0.7	0.189	23/23
70	Homography	0.574	0.4	0.305	23/23
100	Affine	0.452	0.4	0.184	23/23
100	Homography	0.432	0.3	0.231	23/23
120	Affine	0.452	0.4	0.185	23/23
120	Homography	0.228	0.2	0.228	23/23
150	Affine	0.519	0.4	0.180	23/23
150	Homography	0.363	0.1	0.232	23/23

**Table 12:** B-N: all-patch error and descriptive minimum across NCC settings at each size. Errors are all-frame MHPE (m); success has no ground-error cutoff.

Size (px)	Model	All Patches (m)	Selected τ	Minimum (m)	Success
50	Affine	0.345	0.6	0.306	23/23
50	Homography	0.348	0.5	0.315	23/23
70	Affine	0.310	0.5	0.307	23/23
70	Homography	0.318	0.5	0.315	23/23
100	Affine	0.353	0.5	0.306	23/23
100	Homography	0.317	0.2	0.315	23/23
120	Affine	0.351	0.5	0.306	23/23
120	Homography	0.316	0.3	0.314	23/23
150	Affine	0.442	0.5	0.306	23/23
150	Homography	0.317	0.1	0.316	23/23

**Table 13:** B-F: all-patch error and descriptive minimum across NCC settings at each size. Errors are all-frame MHPE (m); success has no ground-error cutoff.

Size (px)	Model	All Patches (m)	Selected τ	Minimum (m)	Success
50	Affine	2.281	0.1	1.757	23/23
50	Homography	4.570	0.5	2.678	11/23
70	Affine	1.576	0.5	0.833	23/23
70	Homography	3.813	0.1	1.580	23/23
100	Affine	1.063	0.4	0.793	23/23
100	Homography	1.151	0.4	1.072	22/23
120	Affine	0.984	0.4	0.795	23/23
120	Homography	1.150	0.2	0.805	23/23
150	Affine	0.883	0.1	0.796	23/23
150	Homography	0.810	0.4	0.805	23/23

**Table 14:** Descriptive NCC statistics pooled over four cameras: 19 vehicle-occluded and 533 clear observations per size. Repeated observations across sizes are not independent samples.

Size (px)	Vehicle Mean NCC	Clear Mean NCC	Vehicle Below 0.5	Clear Below 0.5
50	0.265	0.647	12/19	172/533
70	0.264	0.838	12/19	50/533
100	0.268	0.845	15/19	28/533
120	0.229	0.827	16/19	32/533
150	0.196	0.809	16/19	34/533

**Table 15:** A-N: all-frame hourly comparison. Patch size is 150 px without NCC filtering. H denotes homography.

Method	MHPE (m)	Finite/23	≥10 m Frames
No correction	3.113	23 (baseline)	0
ORB full–H	0.442	23	0
SIFT full–H	0.436	23	0
DISK + MNN full–H	0.421	23	0
DISK + LightGlue full–H	0.420	23	0
Phase–Affine, patch	0.619	23	0
Phase–H, patch	0.455	23	0

**Table 16:** A-F: all-frame hourly comparison. Patch size is 150 px without NCC filtering. H denotes homography.

Method	MHPE (m)	Finite/23	≥10 m Frames
No correction	2.361	23 (baseline)	0
ORB full–H	0.190	23	0
SIFT full–H	0.173	23	0
DISK + MNN full–H	0.178	23	0
DISK + LightGlue full–H	0.172	23	0
Phase–Affine, patch	0.519	23	0
Phase–H, patch	0.363	23	0

**Table 17:** B-N: all-frame hourly comparison. Patch size is 150 px without NCC filtering. H denotes homography.

Method	MHPE (m)	Finite/23	≥10 m frames
No correction	0.457	23 (baseline)	0
ORB full–H	0.312	23	0
SIFT full–H	0.311	23	0
DISK + MNN full–H	0.312	23	0
DISK + LightGlue full–H	0.313	23	0
Phase–Affine, patch	0.442	23	0
Phase–H, patch	0.317	23	0

**Table 18:** B-F: all-frame hourly comparison. Patch size is 150 px without NCC filtering. H denotes homography.

Method	MHPE (m)	Finite/23	≥10 m Frames
No correction	3.545	23 (baseline)	0
ORB full–H	3.712	23	2
SIFT full–H	0.847	23	0
DISK + MNN full–H	0.792	23	0
DISK + LightGlue full–H	0.791	23	0
Phase–Affine, patch	0.883	23	0
Phase–H, patch	0.810	23	0

**Table 19:** Equally weighted 14-camera summary. Relative reduction is 100 × (pixel − ground)/pixel, computed before rounding. Each of the 14 cameras contributes to every row.

Evaluation	Points	Pixel (m)	Ground (m)	Reduction
All-point (in-sample)	190	0.259	0.196	24.4%
Interior LOO	97	0.331	0.318	4.1%
Exterior LOO	93	0.445	0.384	13.5%
Overall LOO	190	0.398	0.348	12.6%

**Table 20:** Count and layout sensitivity on the reviewed dataset. Each strict-subset condition uses ten draws per withheld point. Errors are camera-balanced pixel/ground MHPE; hull is the mean image-area fraction. All finite errors are retained.

Training n	Layout	Cameras	Hull	Pixel/Ground (m)
4	Uniform	14	0.080	8.735/8.735
4	Compact	14	0.027	102.373/102.373
4	Dispersed	14	0.180	1.271/1.271
6	Uniform	14	0.144	0.613/0.828
6	Compact	14	0.077	2.164/2.950
6	Dispersed	14	0.217	0.448/0.474
8	Uniform	11	0.172	0.445/0.466
8	Compact	11	0.096	1.160/1.204
8	Dispersed	11	0.225	0.389/0.390
10	Uniform	7	0.238	0.419/0.396
10	Compact	7	0.138	0.711/0.788
10	Dispersed	7	0.295	0.419/0.388
12	Uniform	5	0.284	0.405/0.369
12	Compact	5	0.179	0.495/0.582
12	Dispersed	5	0.339	0.406/0.337

**Table 21:** Ordinary LOO by image-y tercile. All 14 cameras contribute to each row. Errors are equal-camera MHPE, not pooled-point means.

Image Region	Points	Pixel (m)	Ground (m)
Upper	60	0.567	0.404
Middle	62	0.363	0.357
Lower	68	0.270	0.292

**Table 22:** Affine subset sensitivity at 150 px, no NCC gate. MHPE (m) averages all subsets and frames; the complete subset-level ranges are retained in the analysis tables.

Camera	3 Patches	4 Patches	5 Patches	6 Patches
A-N	1.864	0.624	0.623	0.619
A-F	13,435.702	0.825	0.632	0.519
B-N	0.843	0.484	0.447	0.442
B-F	3.271	1.177	0.970	0.883

**Table 23:** Equal-weight, non-robust model comparison at 150 px using all six centers. All cells have 23 finite evaluations; values are MHPE (m).

Camera	Translation	Similarity	Affine	Homography
A-N	0.506	0.582	0.619	0.681
A-F	0.359	0.359	0.519	9.164
B-N	0.388	0.348	0.442	0.399
B-F	1.272	0.818	0.883	0.929

**Table 24:** Direct robust affine feature baselines versus Phase–Affine on the original 40 frames. Every finite error is included.

Method	Extraction/Support	Finite	MHPE (m)	≥10 m
ORB	Original patch matches	39/40	26.821	7
SIFT	Original patch matches	39/40	4.644	4
DISK + MNN	Original patch matches	40/40	1.391	0
DISK + LightGlue	Original patch matches	39/40	18.788	4
DISK + MNN	Native patch crops	40/40	0.554	0
DISK + LightGlue	Native patch crops	40/40	0.462	0
Phase–Affine	Original patches	40/40	0.392	0

**Table 25:** Strengthened direct-fit benchmark: mean of per-frame median core times from five repetitions after three warm-ups. Feature methods fit individual matches directly with robust affine estimation; DISK uses native-resolution crops. Phase–Affine uses cached reference FFTs. This run differs from the patch-center benchmark in Table 8; compare methods within this table, not absolute times across the two tables.

Method	Finite/40	Mean Core (ms)	MHPE (m)
ORB direct	39	6.417	26.821
SIFT direct	39	28.409	4.644
DISK + MNN direct	40	60.538	0.554
DISK + LightGlue direct	40	106.094	0.462
Phase–Affine	40	9.858	0.392

## Data Availability

Anonymized numerical results and experiment settings are provided in Supplementary Materials S1. The original images and image–GNSS coordinate pairs are company assets and are not available through unrestricted public download. They, together with the associated analysis code, may be provided to reviewers for evaluation and to researchers upon reasonable request to the corresponding author, subject to company authorization and applicable data-protection conditions. The public supplement supports inspection and reaggregation of the reported results; rerunning image registration or calibration from raw observations requires access to the restricted inputs. Third-party software and pretrained weights remain subject to their respective licenses.

## Supplementary Materials

The following supporting information can be downloaded at https://www.mdpi.com/article/10.3390/s26196227/s1: S1, Anonymized numerical results and experiment settings (Supplementary_Materials_S1_20260924.zip), contains S1_static14 (all-point, LOO, perturbation and count/layout results, including subset identifiers and convergence flags), S2_original40 (failure-inclusive accuracy and both runtime benchmarks), S3_hourly92 (size/NCC, subset/model and diagnostic outputs, including pixel-domain patch definitions), and S4_settings (protocols and software/hardware settings). The README, file inventory and aggregation check identify the files and their scope. Images, raw image–GNSS point pairs and geographic coordinates are excluded.

## Appendix A. Complete NCC Threshold Results

Table A1, Table A2, Table A3, Table A4, Table A5, Table A6, Table A7 and Table A8 provide the complete patch-size and signed post-alignment NCC threshold sweep for the four cameras. Columns indicate the minimum NCC threshold; All applies no NCC selection. Rows give the square patch side length. These are the same 400 settings and 9200 frame evaluations summarized in Section 4.6; no additional experiments or exclusions are introduced.

In each cell, the first line is all-frame MHPE (m) and the second is the number of successful estimates with finite evaluation out of 23 frames. Failed estimates use the uncorrected error under the no-update policy; all finite errors, including those at or above 10 m, remain in the average. Success is not defined by a 10 m error cutoff. NCC is a similarity score, not a calibrated probability of success.

Affine uses equal-weight least squares; Homography uses USAC_MAGSAC. Thus, the tables compare these complete fitting configurations. Thresholds are evaluated descriptively, not established as independently validated deployment settings.

### Appendix A.1. A-N

**Table A1:** A-N: Affine. All-frame MHPE and estimation success by patch size and NCC threshold.

Size (px)	All	0.1	0.2	0.3	0.4	0.5	0.6	0.7	0.8	0.9
**50**	2.433 23/23	2.624 22/23	5.867 17/23	4.878 15/23	4.180 13/23	2.254 11/23	2.357 11/23	2.361 11/23	2.361 11/23	2.248 10/23
**70**	1.061 23/23	0.957 23/23	0.823 23/23	0.565 23/23	0.449 23/23	0.449 23/23	0.449 23/23	0.513 23/23	0.552 23/23	0.751 21/23
**100**	0.499 23/23	0.448 23/23	0.448 23/23	0.448 23/23	0.448 23/23	0.448 23/23	0.448 23/23	0.446 23/23	0.538 22/23	0.891 19/23
**120**	0.551 23/23	0.447 23/23	0.447 23/23	0.447 23/23	0.447 23/23	0.448 23/23	0.446 23/23	0.536 22/23	0.536 22/23	1.047 18/23
**150**	0.619 23/23	0.448 23/23	0.448 23/23	0.448 23/23	0.448 23/23	0.447 23/23	0.447 23/23	0.581 22/23	1.256 17/23	1.747 13/23

**Table A2:** A-N: Homography. All-frame MHPE and estimation success by patch size and NCC threshold.

Size (px)	All	0.1	0.2	0.3	0.4	0.5	0.6	0.7	0.8	0.9
**50**	5.592 23/23	8.729 19/23	5.656 12/23	6.131 11/23	4.644 10/23	4.644 10/23	3.972 9/23	4.078 8/23	4.078 8/23	3.186 5/23
**70**	0.988 23/23	1.154 23/23	0.905 23/23	0.573 23/23	0.569 23/23	0.569 23/23	0.569 23/23	0.651 22/23	0.818 20/23	1.148 17/23
**100**	0.463 23/23	0.462 23/23	0.462 23/23	0.462 23/23	0.462 23/23	0.470 23/23	0.473 23/23	0.770 22/23	1.068 21/23	1.557 15/23
**120**	0.458 23/23	0.458 23/23	0.458 23/23	0.458 23/23	0.458 23/23	0.480 23/23	0.569 22/23	0.973 22/23	1.060 21/23	2.098 11/23
**150**	0.455 23/23	0.455 23/23	0.455 23/23	0.455 23/23	0.490 23/23	0.580 22/23	0.968 22/23	1.042 21/23	2.197 15/23	2.458 9/23

### Appendix A.2. A-F

Cell format: MHPE (m) on the first line; successful estimates/23 on the second. All = no NCC selection.

**Table A3:** A-F: Affine. All-frame MHPE and estimation success by patch size and NCC threshold.

Size (px)	All	0.1	0.2	0.3	0.4	0.5	0.6	0.7	0.8	0.9
**50**	0.985 23/23	0.910 22/23	1.015 21/23	0.753 21/23	0.617 21/23	0.601 21/23	0.554 21/23	0.559 21/23	41.425 21/23	42.013 16/23
**70**	0.353 23/23	0.226 23/23	0.195 23/23	0.195 23/23	0.195 23/23	0.195 23/23	0.195 23/23	0.189 23/23	1.715 22/23	2.517 21/23
**100**	0.452 23/23	0.337 23/23	0.312 23/23	0.247 23/23	0.184 23/23	0.187 23/23	0.188 23/23	0.357 23/23	0.927 21/23	0.678 19/23
**120**	0.452 23/23	0.323 23/23	0.248 23/23	0.248 23/23	0.185 23/23	0.187 23/23	1.728 23/23	1.738 23/23	1.964 20/23	2.803 13/23
**150**	0.519 23/23	0.370 23/23	0.370 23/23	0.180 23/23	0.180 23/23	1.873 23/23	1.949 22/23	1.949 22/23	2.187 19/23	3.297 10/23

**Table A4:** A-F: Homography. All-frame MHPE and estimation success by patch size and NCC threshold.

Size (px)	All	0.1	0.2	0.3	0.4	0.5	0.6	0.7	0.8	0.9
**50**	5.139 23/23	2.186 21/23	2.135 21/23	1.449 19/23	29.092 18/23	29.701 18/23	29.695 16/23	2.230 15/23	2.717 13/23	8.908 10/23
**70**	0.574 23/23	0.382 23/23	0.305 23/23	0.305 23/23	0.305 23/23	0.548 23/23	0.548 23/23	2.537 23/23	2.573 19/23	160.473 13/23
**100**	0.432 23/23	0.335 23/23	0.231 23/23	0.231 23/23	0.833 23/23	1.347 23/23	1.350 23/23	0.754 22/23	2.570 17/23	3.063 11/23
**120**	0.228 23/23	0.228 23/23	0.228 23/23	0.230 23/23	0.521 23/23	1.001 23/23	0.715 22/23	0.363 20/23	4.259 16/23	2.376 9/23
**150**	0.363 23/23	0.232 23/23	0.234 23/23	0.235 23/23	0.235 23/23	0.233 22/23	0.362 20/23	0.450 19/23	2.125 11/23	2.926 6/23

### Appendix A.3. B-N

Cell format: MHPE (m) on the first line; successful estimates/23 on the second. All = no NCC selection.

**Table A5:** B-N: Affine. All-frame MHPE and estimation success by patch size and NCC threshold.

Size (px)	All	0.1	0.2	0.3	0.4	0.5	0.6	0.7	0.8	0.9
**50**	0.345 23/23	0.312 23/23	0.307 23/23	0.307 23/23	0.307 23/23	0.307 23/23	0.306 23/23	0.308 23/23	0.308 23/23	0.310 23/23
**70**	0.310 23/23	0.309 23/23	0.309 23/23	0.309 23/23	0.307 23/23	0.307 23/23	0.309 23/23	0.309 23/23	0.327 22/23	0.344 15/23
**100**	0.353 23/23	0.311 23/23	0.311 23/23	0.309 23/23	0.309 23/23	0.306 23/23	0.306 23/23	0.311 22/23	0.340 15/23	0.365 13/23
**120**	0.351 23/23	0.310 23/23	0.310 23/23	0.310 23/23	0.308 23/23	0.306 23/23	0.306 23/23	0.323 16/23	0.461 14/23	0.391 12/23
**150**	0.442 23/23	0.313 23/23	0.313 23/23	0.306 23/23	0.306 23/23	0.306 23/23	0.320 20/23	0.323 16/23	0.346 14/23	0.397 11/23

**Table A6:** B-N: Homography. All-frame MHPE and estimation success by patch size and NCC threshold.

Size (px)	All	0.1	0.2	0.3	0.4	0.5	0.6	0.7	0.8	0.9
**50**	0.348 23/23	0.330 23/23	0.316 23/23	0.316 23/23	0.315 23/23	0.315 23/23	0.315 23/23	0.315 23/23	0.315 23/23	0.385 18/23
**70**	0.318 23/23	0.317 23/23	0.315 23/23	0.315 23/23	0.315 23/23	0.315 23/23	0.315 23/23	0.318 23/23	0.340 21/23	0.365 14/23
**100**	0.317 23/23	0.317 23/23	0.315 23/23	0.315 23/23	0.321 23/23	0.321 23/23	0.338 22/23	0.340 21/23	0.353 15/23	0.388 11/23
**120**	0.316 23/23	0.316 23/23	0.315 23/23	0.314 23/23	0.314 23/23	0.375 23/23	0.378 22/23	0.346 15/23	0.371 13/23	0.427 9/23
**150**	0.317 23/23	0.316 23/23	0.316 23/23	0.336 23/23	0.336 23/23	0.336 23/23	0.350 16/23	0.350 16/23	0.353 14/23	0.434 8/23

### Appendix A.4. B-F

Cell format: MHPE (m) on the first line; successful estimates/23 on the second. All = no NCC selection.

**Table A7:** B-F: Affine. All-frame MHPE and estimation success by patch size and NCC threshold.

Size (px)	All	0.1	0.2	0.3	0.4	0.5	0.6	0.7	0.8	0.9
**50**	2.281 23/23	1.757 23/23	2.197 20/23	1.842 18/23	1.785 18/23	1.842 17/23	1.842 17/23	1.844 17/23	1.951 16/23	2.673 8/23
**70**	1.576 23/23	1.250 23/23	1.019 23/23	0.850 23/23	0.850 23/23	0.833 23/23	0.940 22/23	1.400 18/23	1.464 18/23	1.956 13/23
**100**	1.063 23/23	0.794 23/23	0.794 23/23	0.794 23/23	0.793 23/23	0.794 23/23	0.796 23/23	1.484 18/23	1.844 15/23	2.160 12/23
**120**	0.984 23/23	0.797 23/23	0.797 23/23	0.797 23/23	0.795 23/23	0.796 23/23	1.208 20/23	1.584 16/23	1.595 16/23	2.565 7/23
**150**	0.883 23/23	0.796 23/23	0.796 23/23	0.797 23/23	0.798 23/23	0.836 23/23	1.106 21/23	1.593 16/23	1.721 15/23	2.730 6/23

**Table A8:** B-F: Homography. All-frame MHPE and estimation success by patch size and NCC threshold.

Size (px)	All	0.1	0.2	0.3	0.4	0.5	0.6	0.7	0.8	0.9
**50**	4.570 23/23	10.305 20/23	10.097 16/23	9.953 13/23	10.025 12/23	2.678 11/23	2.842 9/23	2.843 9/23	3.060 7/23	3.545 0/23
**70**	3.813 23/23	1.580 23/23	1.995 20/23	1.687 19/23	1.687 19/23	1.757 18/23	1.961 16/23	1.969 16/23	2.259 14/23	2.674 7/23
**100**	1.151 23/23	1.170 22/23	1.170 22/23	1.170 22/23	1.072 22/23	1.073 22/23	1.311 21/23	2.214 16/23	2.459 13/23	2.657 6/23
**120**	1.150 23/23	0.808 23/23	0.805 23/23	0.805 23/23	0.805 23/23	0.806 23/23	1.913 16/23	2.186 15/23	2.303 11/23	2.778 5/23
**150**	0.810 23/23	0.810 23/23	0.810 23/23	0.806 23/23	0.805 23/23	1.026 23/23	1.517 17/23	1.738 15/23	2.392 9/23	3.004 4/23
